# Components of *Brachypodium distachyon* resistance to nonadapted wheat stripe rust pathogens are simply inherited

**DOI:** 10.1371/journal.pgen.1007636

**Published:** 2018-09-28

**Authors:** Brian Gilbert, Jan Bettgenhaeuser, Narayana Upadhyaya, Melanie Soliveres, Davinder Singh, Robert F. Park, Matthew J. Moscou, Michael Ayliffe

**Affiliations:** 1 CSIRO Agriculture and Food, Clunies Ross Drive, Canberra, ACT, Australia; 2 The Sainsbury Laboratory, Norwich Research Park, Norwich, United Kingdom; 3 University of Sydney, Plant Breeding Institute, Cobbitty, NSW, Australia; 4 University of East Anglia, Norwich Research Park, Norwich, United Kingdom; Danforth Center, UNITED STATES

## Abstract

Phytopathogens have a limited range of host plant species that they can successfully parasitise ie. that they are adapted for. Infection of plants by nonadapted pathogens often results in an active resistance response that is relatively poorly characterised because phenotypic variation in this response often does not exist within a plant species, or is too subtle for genetic dissection. In addition, complex polygenic inheritance often underlies these resistance phenotypes and mutagenesis often does not impact upon this resistance, presumably due to genetic or mechanistic redundancy. Here it is demonstrated that phenotypic differences in the resistance response of *Brachypodium distachyon* to the nonadapted wheat stripe rust pathogen *Puccinia striiformis* f. sp. *tritici* (*Pst*) are genetically tractable and simply inherited. Two dominant loci were identified on *B*. *distachyon* chromosome 4 that each reduce attempted *Pst* colonisation compared with sib and parent lines without these loci. One locus (*Yrr1*) is effective against diverse Australian *Pst* isolates and present in two *B*. *distachyon* mapping families as a conserved region that was reduced to 5 candidate genes by fine mapping. A second locus, *Yrr2*, shows *Pst* race-specificity and encodes a disease resistance gene family typically associated with host plant resistance. These data indicate that some components of resistance to nonadapted pathogens are genetically tractable in some instances and may mechanistically overlap with host plant resistance to avirulent adapted pathogens.

## Author summary

Plant pathogens are specialists and can colonise only a limited number of plant species (hosts). Pathogen infection of a plant that is not a host of the disease often results in an active plant defense response. This poorly characterised defense response is durable as phytopathogens rarely successfully colonise new hosts. The ability to transfer this resistance to host plants would be highly beneficial in protecting crops from disease. However, this resistance has been difficult to genetically dissect as all members of a plant species are resistant. Here we show that some accessions of the model grass *Brachypodium distachyon* show some variation in their ability to suppress colonisation by the wheat stripe rust pathogen *Puccinia striiformis*. Brachypodium is not a host species of wheat stripe rust disease and no accessions are fully susceptible, however, some allow more pathogen growth than others. We have exploited these relatively subtle phenotypic differences to genetically dissect this difference in resistance and identified two Brachypodium loci that contribute increased resistance to the nonadapted wheat stripe rust pathogen.

## Introduction

Only a limited number of phytopathogen species are adapted to paracitise a given plant species. The numerous phyopathogens unable of colonising a plant species (nonadapted) are often suppressed by an active plant resistance response upon challenge [1, 2, 3, 4]. Plant resistance against nonadapted pathogens is considered durable as pathogen colonisation of new plant host species is rare, at least over short evolutionary time frames [5, 6]. However, nonadapted pathogen infection can result in a variety of outcomes varying from complete plant immunity, to limited pathogen colonisation and reproduction [7].

Mechanistically resistance to nonadapted pathogens can range from a basic physical or chemical incompatibility between a pathogen and potential plant host, to active recognition of pathogen challenge by the nonhost plant leading to a complex defense response [1, 8, 9, 10]. Current molecular models of active defense invoke similar mechanisms used to protect host plants against avirulent adapted pathogens, these being phytoalexin defences, callose deposition, reactive oxygen production and cell death in some instances [9, 10, 11].

Plant recognition of pathogen attack occurs by plant pathogen or damage associated molecular pattern (PAMP/DAMP) receptors [12, 13, 14]. Adapted pathogens repress signalling by these receptors by the introduction of pathogen effector molecules into infected plant cells. Some members of a host plant species can recognise specific pathogen effector molecules or their enzymatic activity and then activate a defense response (ie. effector triggered immunity) [8]. Recognition of nonadapted pathogens is likely coupled with an inability of the nonadapted pathogen effector complement to effectively supress PAMP/DAMP signalling [4, 8, 10, 15]. Effectors from nonadapted pathogens can also be recognised by incompatible plant species in some instances thereby including effector triggered immunity, a typical host/pathogen defense response, in nonadapted pathogen resistance [16, 17, 18, 19, 20].

At a molecular level resistance to nonadapted pathogens has often been difficult to investigate. Within a species this resistance often shows no, or very subtle, phenotypic variation negating classical mapping and positional cloning strategies [21, 22, 23]. In some cases variation can be observed but controlled by multiple genes, each with small additive affects making isolation of the underlying QTL difficult [24, 25, 26, 27, 28, 29]. However, mutagenesis and virus induced gene silencing, usually in model species, has had some success [4]. For example, the isolation of the Arabidopsis *PEN* genes by mutagenesis has provided insights into the mechanistic basis of penetration resistance faced by nonadapted mildew pathogens [30, 31, 32]. However, once these penetration barriers are overcome additional mutations are required for increased nonadapted pathogen development consistent with a multiplicity of resistance barriers being faced by an invading pathogen [31, 33, 34].

Increasing phylogenetic distance between two plant species reduces their likelihood of both being hosts to the same morphological stage of a pathogen species [35]. This phylogenetic distance also appears to influence the extent of nonadapted pathogen growth on nonhost plants [10, 11]. Previously we examined rice resistance to the nonadapted cereal rust pathogens *Puccinia graminis* f. sp. *tritici*, *P*. *triticina*, *P*. *striiformis* f. sp. *tritici* and *P*. *hordei*. Each pathogen was capable of infecting rice and on occasion colonising hundreds of mesophyll cells, however sporulation was never observed [22]. Only subtle genetic variation in infection phenotypes was observed between different rice lines and mutagenesis was unsuccessful in identifying plants with perturbed resistance to these nonadapted fungal species [22]. The flax rust pathogen (*Melampsora lini*) of the dicotyledonous plant species *Linum ussitatissimum* (flax) was even less able to colonise rice, rarely being able to identify stomates on the leaf surface to enter the leaf [22]. These observations are consistent with increased phylogenetic distance between host and nonhost plant species being associated with a reduced ability of nonadapted pathogens to colonise. Similar poor colonisation was observed upon challenge of *Arabidopsis thaliana* with the wheat leaf rust pathogen, *P*. *triticina* [27].

The model temperate grass *Brachypodium distachyon* is more closely related to cereal rust hosts like wheat and barley than rice [36]. Unlike rice, *B*. *distachyon* is also parasitized by a rust disease pathogen, *P*. *brachypodii* [37, 38]. Also unlike rice, the nonadapted cereal rust pathogens *P*. *graminis*. f. sp. *tritici* and *P*. *striiformis* f. sp. *tritici* are capable of occasionally producing very small sporulating uredinia on *B*. *distachyon* [38, 39, 40, 41]. In contrast, the rust pathogen (*Puccinia emaculata*) of *Panicum virgatum* (switchgrass) shows limited infection of *B*. *distachyon* [23] again suggesting that phylogenetic relatedness of host and nonhost species influences nonadapted pathogen infection outcomes.

Those *B*. *distachyon* accessions that allow the most growth of cereal rust pathogens are nonetheless still highly resistant when compared with a susceptible wheat genotype or even some wheat genotypes that are classified as having resistance to rust disease. In fact, on most *B*. *distachyon* accessions no macroscopic symptoms are observed and microscopic analyses show limited infection by these nonadapted pathogens with sites usually consisting of an appressorium, substomatal vesicle and a few infection hyphae [41]. *B*. *distachyon* accessions challenged with cereal rust pathogens responded with an active defense response that involves callose deposition and H_2_O_2_ accumulation, whereas autofluorescent cell death is relatively uncommon [40, 41]. *P*. *graminis*. f. sp. *tritici* and *P*. *striiformis* f. sp. *tritici* are therefore not adapted to parasitise *B*. *distachyon*, however for some accessions the resistance to these pathogens is not as restrictive as that observed on rice.

**Fig 1:**
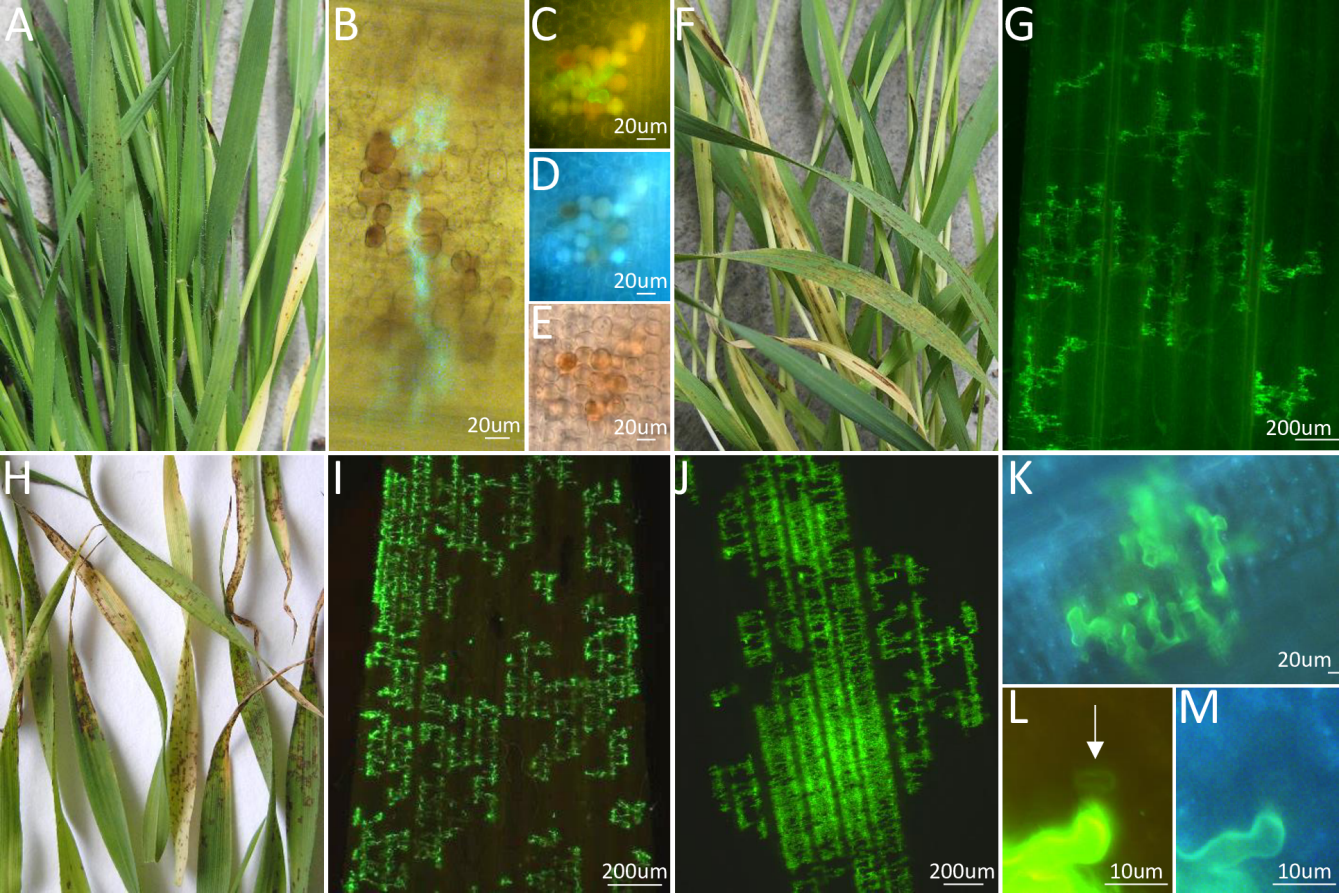
*Pst* infection phenotypes on *B*. *distachyon* accessions. A) *B*. *distachyon* accession BdTR10h infected with *Puccinia striiformis* f. sp. *tritici* (*Pst*) isolate 104 E137 A–. B-C) *Pst* pathotype 104 E137 A–infection sites on BdTR10h leaf tissue stained with wheat germ agglutinin conjugated to fluorescein isothiocyanate (WGA-FITC), a lectin-fluorophore that specifically binds chitin. Green fungal infection hyphae can be seen ramifying between mesophyll cells, some of which contain brown pigmentation. D-E) The same *Pst* infection site shown in C). Some autofluorescent mesophyll cells are observed under UV light (D) while brown pigmentation is seen under bright light (E). F) Macroscopic infection symptoms of *Pst* pathotype 104 E137 A–on *B*. *distachyon* accession Tek-4 showing obvious striped lesions. G) Staining of *Pst* pathotype 104 E137 A–infection hyphae in a Tek-4 leaf. Large infection sites are observed. H) Macroscopic infection phenotype of *Pst* pathotype 104 E137 A–on *B*. *distachyon* accession Bd21. I) Growth of infection hyphae of *Pst* pathotype 104 E137 A–throughout a Bd21 leaf. Tissue was stained with WGA-FITC and observed under blue light. J) Extensive growth of *Pst* pathotype 104 E137 A–in leaf tissue of a yrr2 progeny plant derived from plant F_4-_56. K) Limited *Pst* pathotype104 E137 A–development on leaf tissue of a Yrr2 progeny plant derived from plant F_4_-56. The leaf sample was stained with WGA-FITC and photographed under UV light. No autofluorescent cell death was observed. L-M) A *Pst* pathotype 104 E137 A–haustorium (arrowed) growing in leaf tissue from a Yrr2 F5 plant derived from F_4_ plant 56 (L). The haustorium infected plant mesophyll cell has not undergone autofluorescent cell death when viewed under UV light (M). All images were taken approximately 21 days post infection (dpi).

Previously we developed two *B*. *distachyon* genetic mapping families that segregate for differential outcomes to *P*. *striiformis* f. sp. *tritici* (*Pst*) challenge [41]. In each family one parent (BdTR10h and BdTR13k, respectively) had highly restricted *Pst* growth with infection sites consisting of a substomatal vesicle and a few infection hyphae that colonised a limited number of mesophyll cells with haustoria (Fig 1) [41]. In contrast, the other parent in the cross (Tek-4 and Bd21, respectively) was more extensively infected by *Pst* with hundreds of mesophyll cells colonised (Fig 1). Occasionally these latter *Pst* infection sites produced tiny sporulating uredinia, although only after long (3–4 week) post infection time periods and specific growth conditions [41]. In a cross between *B*. *distachyon* accessions BdTR10h and Tek-4, these differential infection outcomes segregated as a 3:1 ratio with restricted infection being inherited as a single dominant gene [41]. In the second mapping family, BdTR13k x Bd21, highly restricted pathogen growth predominated amongst progeny and segregation data most closely supported a 1 dominant, 1 recessive gene model, or alternatively a two dominant, linked gene model, with each gene independently suppressing pathogen growth [41].

In this report we undertake detailed fine mapping studies and demonstrate that a single dominant locus highly restricts *Pst* growth in a BdTR10h x Tek-4 family, whereas two dominant linked loci independently confer a similar phenotype in a BdTR13k x Bd21 family with one locus being nearly identical to that present in the former family. These data show that in *B*. *distachyon* some components of resistance to the nonadapted pathogen *Pst* are genetically simply inherited. These resistance components are superimposed on a background of *B*. *distachyon* incompatibility to colonisation by these nonadapted cereal rust pathogens.

## Results

### The *Yrr1* gene segregates in each mapping family

Previous analysis of *B*. *distachyon* accessions BdTR10h and Tek-4 showed that macroscopically the former accession produced small brown lesions in response to challenge with *Pst* pathotype 104 E137 A–, whereas large, striped lesions were produced on Tek-4 plants [41] (Fig 1A and 1F). Each small brown lesion on BdTR10h corresponded to a small *Pst* infection site usually comprising several infection hyphae, occasional haustoria and limited numbers of weakly autofluorescent mesophyll cells, some of which contained nonfluorescent, brown pigments [41] (Fig 1B–1E). In contrast, larger infection sites that colonised hundreds of mesophyll cells and formed striped lesions growing parallel to leaf veins occurred on Tek-4 plants (Fig 1G). Infection of a BdTR10h x Tek-4 mapping family with *Pst* pathotype 104 E137 A–demonstrated that amongst 213 F_2_ plants, 159 allowed very little pathogen development while 54 plants showed more extensive pathogen growth [41] (S1 Fig). This single dominant locus (X^2^ 3:1, p = 0.90) that restricted *Pst* development was designated *Yrr1* (***Y****ellow* ***r****ust* ***r****esistance gene* ***1***).

**Fig 2:**
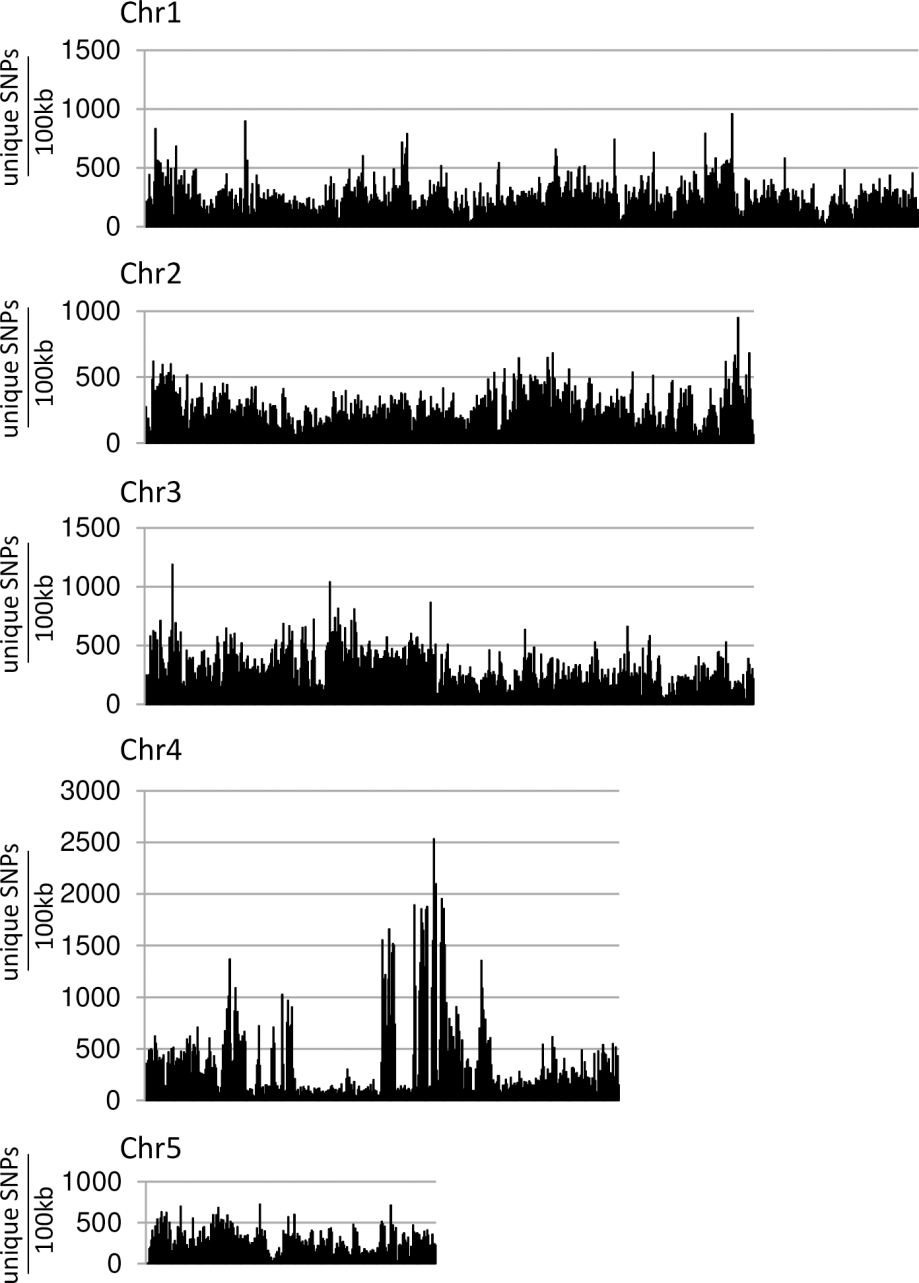
SNP analysis of the BdTR10h x Tek-4 *Yrr1* DNA bulk. SNPs were identified relative to the Bd21 reference genome. Those SNPs that were unique to the *Yrr1* DNA pool (ie. absent in the *yrr1* DNA bulk sequence) were mapped to Bd21 reference chromosome sequences. Each data point is the number of unique SNPs identified relative to a 100 kb window of the reference chromosome sequence. A large peak of SNPs unique to the resistant DNA bulk was detected at 29Mb on chromosome 4.

Genomic DNA deep sequencing was coupled with bulk segregant analysis [42] to rapidly localise the *Yrr1* locus. DNA samples from 153 BdTR10h x Tek-4 F2 plants that showed restricted pathogen growth were pooled (*Yrr1/Yrr1* and *Yrr1/yrr1*) while a second DNA pool (*yrr1/yrr1*) was produced from 49 F_2_ plants that were more extensively colonised by *Pst*. Each DNA pool was sequenced (40x coverage, 100 bp paired end reads) and compared with the Bd21 reference genome [43]. Relative to the Bd21 genome 4,845,783 SNPs were identified in the *Yrr1* DNA pool while 5,014,386 SNPs were identified in the *yrr1* DNA bulk. SNPs unique to the *Yrr1* DNA pool (ie. absent in the *yrr1* pool) were identified and then aligned relative to the Bd21 reference genome sequence. The number of SNPs present in 100 kb intervals across the length of each Brachypodium chromosome was determined and graphed for these unique *Yrr1* SNPS (Fig 2). A background of approximately 500 SNPs per 100 kb window was observed along each chromosome that was presumably due to sequencing errors and sequence misalignment (Fig 2). However, a substantial increase in the number of unique SNPs present in the *Yrr1* DNA pool was observed in the 27–30 Mbp region of chromosome 4 with a single 100 kb region containing in excess of 2,500 SNPs that were absent in the *yrr1* pool (Fig 2). These data locate *Yrr1* within the 27–30 Mbp interval of chromosome 4.

To confirm the chromosome 4 map location of *Yrr1* four polymorphic PCR sequencing markers (SNP29017600, SNP29419400, SNP29800350 and SNP30968800 (S1 Table) that spanned the region of interest were used to genotype 212 F2 plants that had been phenotyped for *Pst* infection (note SNP names indicate approximate nucleotide positions on the *B*. *distachyon* Bd21 chromosome 4 reference sequence version 1.2 (http://www.plantgdb.org/BdGDB)). Twenty six plants were identified with recombination events between markers SNP29017600 and SNP30968800. These plants were further genotyped with additional markers throughout the region (S1 Table) and two Yrr1 plants (F_2_ lines 113 and 172) were identified as recombinant in a 400 kb interval between markers SNP29419400 and SNP29800350.

**Fig 3:**
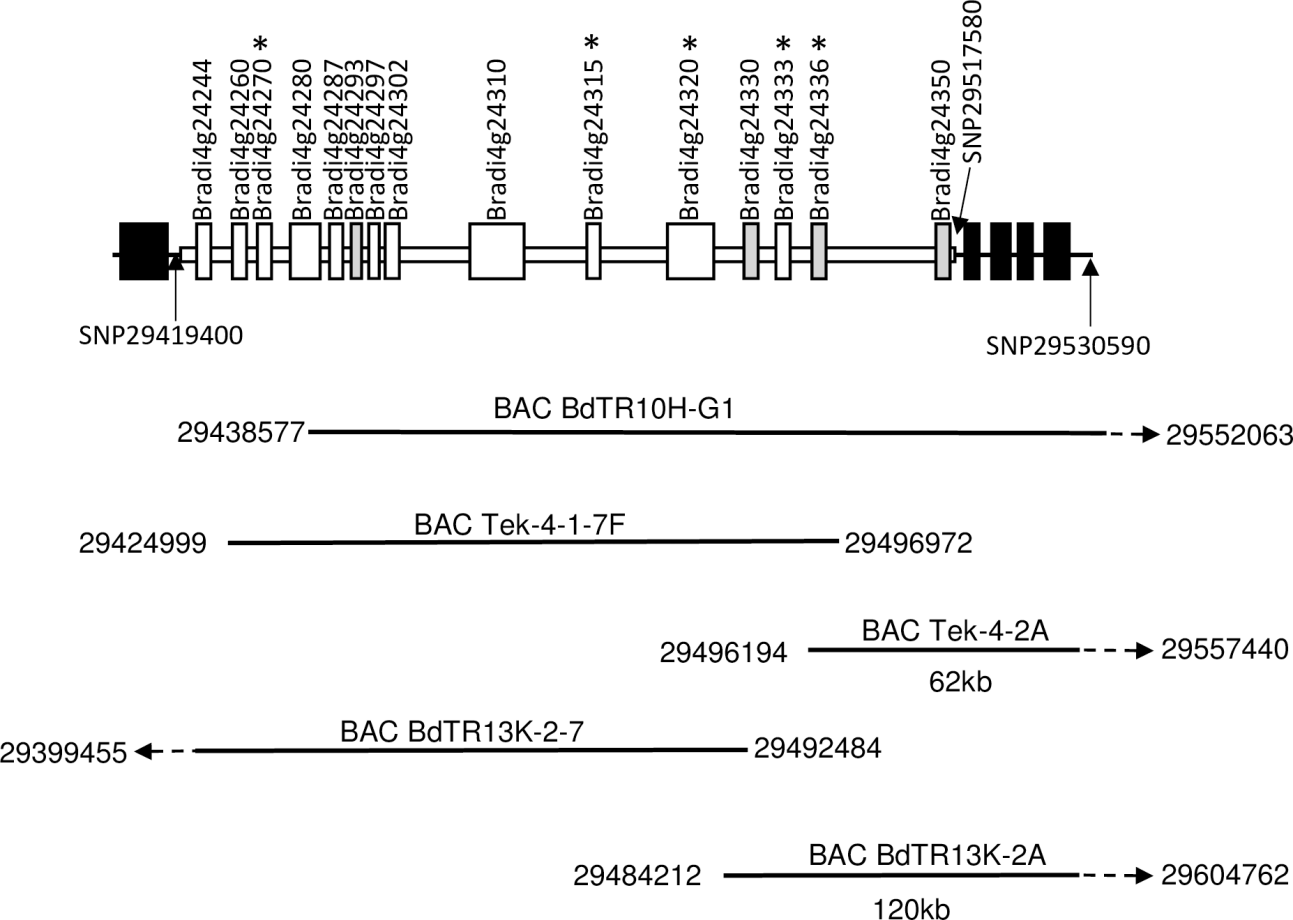
The *Yrr1* locus. Location of BAC clones isolated from the *Yrr1* locus. The upper image (line with black and white boxes) depicts genes annotated in the Bd21 reference genome sequence. White boxes indicate genes present in the *Yrr1* interval while black boxes indicate genes flanking *Yrr1*. Grey boxes indicate genes not included in the *Yrr1* haplotype analysis while genes marked with an asterisk were identified as *Yrr1* candidates. Gene annotation is as per the JGI Brachypodium reference genome sequence version 3. Lines beneath indicate the positions of sequenced BAC clones isolated from BdTR10h, Tek-4 and BdTR13k genomic DNAs. Numbers correspond to nucleotide positions of the Bd21 chromosome 4 reference sequence. The position of critical SNP markers defining the *Yrr1* interval are labelled and indicated by arrows.

These two critical recombinants and their F_3_ progeny were genotyped with 14 SNP markers throughout this 400 kb interval (S1 Table: SNP29419400—SNP29530590). *Pst* infection indicated that the Yrr1 phenotype segregated in each F_3_ family (S2 Fig). Genotyping and pathology analysis of F_3_ individuals from these two families confirmed that *Yrr1* is located between markers SNP29419400 and SNP29517580 (Fig 3). Fifteen genes are annotated in this 100 kb region of the Bd21 reference genome (JGI *B*. *distachyon* genome v 3, Bradi4g24244 –Bradi4g24350) none-of-which encode proteins such as nucleotide binding site, leucine rich repeat proteins (NLR) proteins which are often associated with disease resistance loci (S2 Table).

**Fig 4:**
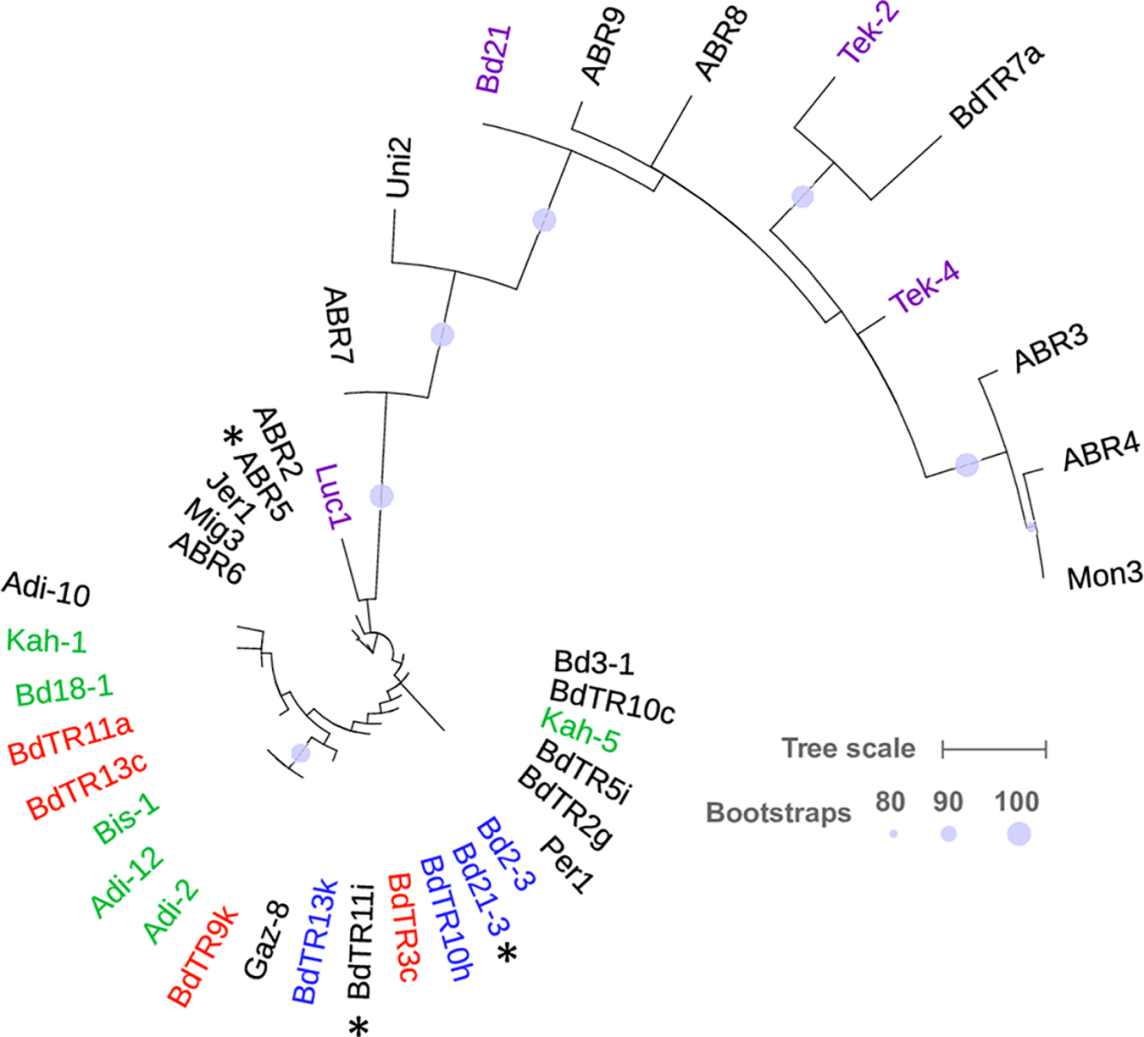
Two distinct haplotypes exist at the *Yrr1* locus. The phylogenetic tree was constructed using maximum likelihood with model GTRCAT and rapid hill-climbing mode. Scale bar units are substitutions per site, with the bar representing 0.1 (based on polymorphic sites only). Bootstrap support was generated using 2,000 bootstrap runs, with only support above 80% shown in the figure. For simplicity a single representative is shown for clades containing multiple accessions (marked with an asterisk). A more complete phylogeny is shown in S3 Fig. Red labelling indicates accessions that were immune to Australian *Pst* isolate 104 E137 A-, while those in green were immune to UK isolate 08/501. These accessions were tested with a single *Pst* isolate only. Accession labelled blue were immune to both isolates [41]. Those accessions labeled purple allowed more extensive *Pst* growth although Luc1 and Tek-2 were tested with only the UK and Australian isolate, respectively.

Two SNP markers from the *Yrr1* interval, SNP29419400 and SNP29530590, were used to PCR screen BAC libraries of *B*. *distachyon* accessions BdTR10h, BdTR13k and Tek-4. BAC clones were isolated for each genotype and sequenced (Fig 3). Only a single BAC clone was isolated from accession BdTR10h which did not cover the 98 kb *Yrr1* interval in its entirety, missing approximately 19 kb of sequence adjacent to SNP29419400. Two Tek-4 BAC clones from the *yrr1* locus were sequenced that covered the interval apart from missing 6kb of sequence in juxtaposition to SNP29419400 (Fig 3). The 80 kb of *Yrr1* locus sequence available from BdTR10h showed near sequence identity (99%) with the equivalent region from BdTR13k. In contrast more sequence variation occurred between the *yrr1* locus of Bd21 and Tek-4 and both of these sequences and the conserved *Yrr1* locus present in BdTR10h and BdTR13k. This variation was further confirmed by the haplotype analysis described below with genetic distances shown in Fig 4. From these data it is appears that the *Yrr1* locus is present in both BdTR10h and BdTR13k as part of a highly conserved genomic segment. The presence of *Yrr1* in BdTR13k was further confirmed by the genetic analysis described below.

### Haplotype analysis identifies 5 gene candidates at the *Yrr1* locus

To further understand the haplotype diversity at the *Yrr1* locus the nucleotide variation in a diverse panel of 47 resequenced *B*. *distachyon* accessions was examined. Alignment of reads to the Brachypodium Bd21 reference genome spanning the region encompassing Bradi4g24244 to Bradi4g24350 identified 104 polymorphic nucleotide sites across 14.7 kb of coding sequence derived from 11 genes at the *Yrr1* locus (S3 Table). Phylogenetic analysis using maximum likelihood (Fig 4) and neighbor joining (S3 Fig) identified trees with similar topologies, which established two distinct haplotypes (Yrr1.h1 and Yrr1.h2). The immune accessions BdTR10h and BdTR13k contain Yrr1.h1 whereas Bd21 and Tek-4, which are more extensively colonized by *Pst*, contain Yrr1.h2.

While 15 genes are annotated at the *Yrr1* locus, four genes (Bradi4g24293, Bradi4g24330, Bradi4g24336, Bradi4g24350) were excluded from the haplotype analysis due to the presence of indels in the coding sequence that resulted in a frame shift or SNPs that led to the generation of an early stop codon in some accessions (S4 Table). Bradi4g24330 and Bradi4g24350 are pseudogenes in accession BdTR10h (Yrr1) while Bradi4g24293 is a pseudogene in BdTR13k (Yrr1) (S4 Table). These three genes are therefore not *Yrr1* candidates and were not considered further. Bradi4g24336, while a pseudogene in some accessions, encodes an intact ORF in the immune parents and was therefore analysed independently as described below.

Previously we had tested many of the resequenced accessions with either an Australian (104 E137 A–) or UK (08/501) *Pst* isolate, both of which are recognized by *Yrr1* [41] (Fig 4). SNPs from 10 of the 11 genes used in the haplotype analysis were compared between immune plants known to contain *Yrr1* ie. BdTR10h, BdTR13k and ABR6 (see accompanying paper by Bettgenhaeuser et al. for ABR6 genotyping) and those that show more extensive *Pst* colonization and therefore do not contain *Yrr1* ie. Bd21, Tek-4, Tek-2 and Luc1 (Fig 4). Bradi4g24297 was excluded from further analysis as it encodes a reverse transcriptase protein common to plant retrotransposable elements and so is an unlikely *Yrr1* candidate.

Only three genes (Bradi4g24270, Bradi4g24320, Bradi4g24333) contain SNPs that are uniquely polymorphic between the two resistance classes (S5 Table, highlighted in blue). These unique SNPs result in changes in the predicted proteins of Bradi4g24270 and Bradi4g24320 making these genes *Yrr1* candidates (Table 1). For Bradi4g24333 this differential SNP is a synonymous change. However, for both this gene and Bradi4g24315 (S4 Fig) no identical protein is encoded by an immune and more susceptible accession therefore making both these genes *Yrr1* candidates (Table 1). For all the remaining genes at the locus at least one immune and one more susceptible accession encode identical proteins thereby excluding these genes as *Yrr1* (S5 Table, highlighted in grey).

**Table 1:** Amino acid differences in Yrr1 candidate proteins.

Gene	Protein size	a.a. posn.	BdTR10h Yrr1	BdTR13k Yrr1	ABR6 Yrr1	Bd21 yrr1	Tek-2 yrr1	Tek-4 yrr1	Luc1 yrr1	Predicted product
Bradi4g24270	563 a.a	92	D	D	D	D	D	D	N	glyoxal oxidase
		143	*T	**T**	**T**	**N**	**N**	**N**	**N**	
#Bradi4g24315	197a.a									sentrin/SUMO specific protease
Bradi4g24320	556 a.a	62	**L**	**L**	**L**	**H**	**H**	**H**	**H**	CBS domain protein
		147	S	S	S	S	L	S	S	
		552	**I**	**I**	**I**	**K**	**K**	**K**	**K**	
Bradi4g24333	120 a.a	10	S	S	S	S	S	S	F	unknown hypothetical protein
		20	C	C	C	C	C	Y	C	
		22	S	S	S	R	R	R	S	
		29	M	M	K	M	M	M	M	
		33	P	P	P	L	L	L	P	
		68	G	G	G	R	G	G	G	
		74	R	R	R	R	R	R	I	
#Bradi4g24336	718 a.a									Unknown protein with DUF4220 domain

* polymorphisms unique to Yrr1 and yrr1 classes shown in bold.

# See S4 Fig and S6 Table for Bradi4g24315 and Bradi4g24336 protein comparisons.

Additional plants previously shown to be immune to *Pst* [41] (Fig 4) are also shown in S5 Table and, where sequence was determined, each maintains all highlighted (blue) SNPs unique to the *Yrr1* genotype, with the exception of the polymorphism in Bradi4g24270 in accession Adi2. As *Pst* immunity has not been shown to segregate with the *Yrr1* locus in these latter plants they could contain a nonfunctional *Yrr1* locus with immunity provided by an alternative gene and hence Bradi4g24270 remains a candidate. None-the-less, these additional accessions show a co-relation between *Pst* immunity and the SNPs unique to *Yrr1*.

Independent analysis of Bradi4g24336 showed that *Yrr1* lines BdTR13k, BdTR10h and ABR6 all encode identical coding sequences and consequently identical predicted proteins (S6 Table). Proteins encoded by Bd21 (yrr1) and Luc1(yrr1) show approximately 97% amino acid identity to each other and the protein encoded by the *Yrr1* genotypes. In both Tek-2 and Tek-4, Bradi4g24336 has a mutated gene start codon with no alternative start site in immediate proximity suggesting that it is a pseudogene in these accessions. Seven SNPs and three amino acids are unique to genes and proteins encoded by the more susceptible genotypes including the Tek-2 and Tek-4 pseudogenes (S6 Table). The Bradi4g24336 gene, which encodes a protein of unknown function containing a DUF4220 domain, is therefore a *Yrr1* candidate (Table 1).

**Fig 5:**
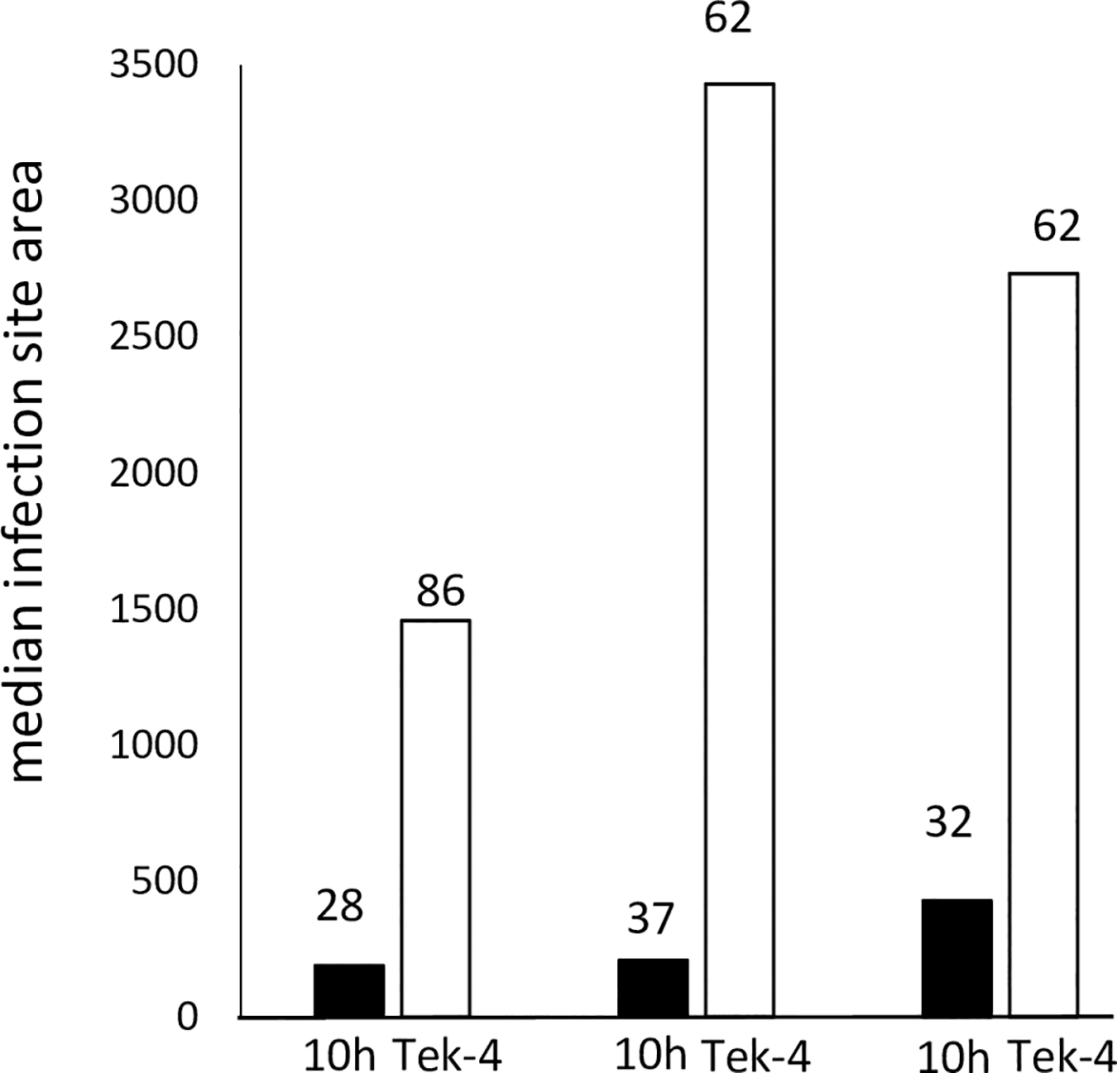
The *Yrr1* locus provides resistance to multiple *Pst* pathotypes. BdTR10h and Tek-4 were infected with three *Pst* pathotypes, these being from left to right isolate 110 E143 A+, 134 E16 A+ and 108 E141 A-, respectively. In each experiment the median infection site area was determined by staining infected leaf tissue with WGA-FITC and microscopically measuring individual infection sites approximately 21 dpi. Numbers above each column indicate the number of infection sites measured in each case. The minimum criteria to be counted as an infection site were spores that had germinated and developed at least appressoria and substomatal vesicles. Significantly greater *Pst* growth occurred on Tek-4 for all three isolates (Mann Whitney U-test, p < 0.05).

To test the *Pst* race-specificity of *Yrr1*, BdTR10h (*Yrr1/Yrr1*) and Tek-4 (*yrr1/yrr1*) were challenged with a further three *Pst* pathotypes that represent the genetic diversity of this pathogen in Australia. Twenty one days post infection leaves from each accession were stained with wheat germ agglutinin conjugated to fluorescein isothiocyanate (WGA-FITC), a lectin-fluorophore that specifically binds fungal chitin [22, 41]. Rust infection sites were microscopically measured on each accession and median infection site areas calculated. Each *Pst* isolate showed greatly reduced fungal growth on BdTR10h compared with Tek-4 (Mann Whitney U-test, p < 0.05), providing no evidence for race-specificity being conferred by *Yrr1* (Fig 5).

### The *Yrr2* gene

Previously we reported that infection of *B*. *distachyon* accession BdTR13k with *Pst* pathotype 104 E137 A–resulted in very limited pathogen growth, similar to that observed on BdTR10h plants, although no brown lesions developed on the leaf surface and no macroscopic symptoms were evident ie. the plants showed macroscopic immunity [41]. In contrast, more extensive *Pst* growth occurs on Bd21, similar to that observed on Tek-4, with larger infection sites corresponding to obvious macroscopic lesions on leaves (Fig 1H and 1I). Amongst 316 F_4_ plants in a BdTR13k x Bd21 mapping family 228 individuals were macroscopically immune to *Pst* infection while 88 individuals showed obvious lesions that were confirmed as *Pst* infection sites by microscopy [41]. These data do not fit the segregation ratios expected of a single dominant gene or two unlinked dominant genes in an F4 family.

**Fig 6:**
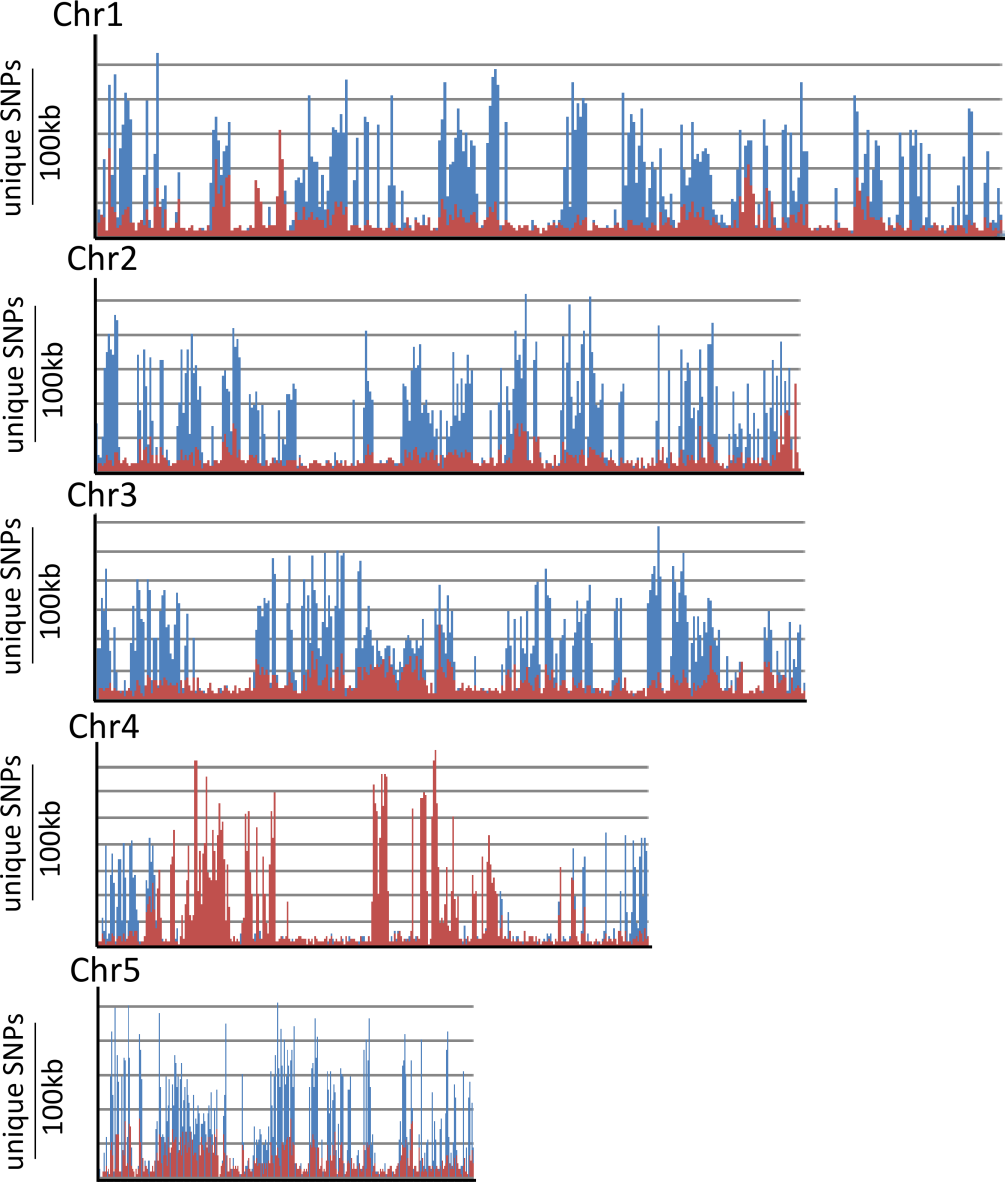
SNP analysis of BdTR13k x Bd21 bulked DNA sequences. SNPs were mapped relative to the Bd21 reference genome chromosome sequences with each datapoint showing the number of SNPs identified in a 100 kb interval of sequence. Datapoints in blue indicate SNPs found between both DNA bulk sequences and the Bd21 reference genome (ie. essentially showing the distribution of SNPs between the BdTR13k and Bd21 genome). Superimposed on the blue graph are SNPS that were unique to the DNA bulk derived from immune plants (ie. absent in the DNA bulk derived from plants showing the greatest *Pst* growth). Two distinct red peaks in the immune DNA bulk data, at 8 Mb and 29 Mb on chromosome 4, can be seen that obscure underlying blue peaks.

To identify loci conferring resistance in this population, deep sequencing (40x coverage) of F_3_ DNA bulks derived from 34 immune plants and 20 plants that showed macroscopic *Pst* symptoms was performed. Relative to the Bd21 genome 915,562 SNPs were identified in the immune pool and 781,411 SNPs in the second pool. In this DNA pooling experiment all SNPs identified were theoretically derived from the BdTR13k parent as Bd21 was the reciprocal parent in this cross and its genome was used for SNP identification. The immune DNA pool was potentially derived from both heterozygous and homozygous plant genotypes. Two distinct peaks of SNPs were identified in the immune DNA bulk that were under-represented in DNAs from plants showing *Pst* symptoms (Fig 6). Both SNP peaks were located on the chromosome 4 reference sequence with one peak at 29Mb coinciding with *Yrr1*, consistent with the sequence similarity with BdTR10h in this region described above, while a second locus was identified at 8Mb on the other arm of chromosome 4. This locus was subsequently named *Yrr2*.

To further refine the *Yrr2* locus, F_3_ DNA samples from 52 plants (32 immune, 20 with more *Pst* growth) used in the DNA bulking process were genotyped with a series of SNP markers spanning the 8 Mb region (S7 Table; SNP6716730 through to SNP11475500, excluding CAPs and KASP marker) and also for two SNP markers that defined the *Yrr1* interval (S1 Table, SNP29419400 and SNP29530590; Fig 3). All 20 plants that showed macroscopic symptoms (ie. that were more extensively colonised) were homozygous for Bd21 SNPs using the two *Yrr1* flanking markers as well as for SNP markers from SNP7761780 through to SNP9888760 (S7 Table). Markers immediately outside this region (S7 Table, SNP7733430 and SNP9983700) contained SNPs from the immune BdTR13k parent in some of these more extensively *Pst* colonised plants. These data suggested that *yrr2* is located between SNP7733430 and SNP9983700. Genotyping of the 32 immune F_3_ plants showed that 30 contained at least one *Yrr1* allele that conferred immunity. The remaining two immune plants (*yrr1/yrr1*) contained BdTR13k SNPs across the SNP7733430 to SNP9983700 interval consistent with a second locus that also suppressed *Pst* colonisation, *Yrr2*, being present in these latter two plants.

F_5_ families from the BdTR13k x Bd21 cross were screened to identify segregating families monogenic for either *Yrr1* or *Yrr2*. Two F5 families were identified, one from plant F_4_-93 (*Yrr1/yrr1; yrr2*/*yrr2*) and the other from plant F_4_-56 (*yrr1*/*yrr1; Yrr2*/*yrr2*). Repeated testing of F_5_ progeny showed that *Pst* immunity segregated as a single dominant gene in each family (examples shown in S5 Fig). Having developed a monogenic family segregating for *Yrr2* microscopic analyses of *Pst* infection phenotypes were undertaken on F_5_ progeny. In Yrr2 plants *Pst* infection sites were again restricted to just a few limited infection hyphae with occasional haustoria (Fig 1K–1M). Autofluorescent cell death was not common (Fig 1K and 1M). In contrast, *yrr2* sib plants had more extensive *Pst* colonisation similar to that observed on the Bd21 parent (Fig 1J). The Yrr2 histological phenotype therefore appeared very similar to that conferred by Yrr1. Plants that contained both *Yrr1* and *Yrr2* did not show enhanced suppression of rust infection compared with plants containing either gene singularly indicating that these two resistance genes do not function additively.

However, in contrast to *Yrr1*, when F_5_ progeny of plant F_4_-56 (*yrr1/yrr1*; *Yrr2/yrr2*) were challenged with a second *Pst* pathotype, 134 E16 A+, all seedlings developed macroscopic symptoms regardless of the *Yrr2* genotype. Microscopic analysis of lesions on these plants confirmed extensive fungal colonisation on both *Yrr2* and *yrr2* genotypes similar to that observed on the Bd21 parent. These data indicate that the *Yrr2* gene shows race-specificity to *Pst* and does not restrict growth of *Pst* pathotype 134 E16 A+.

**Fig 7:**
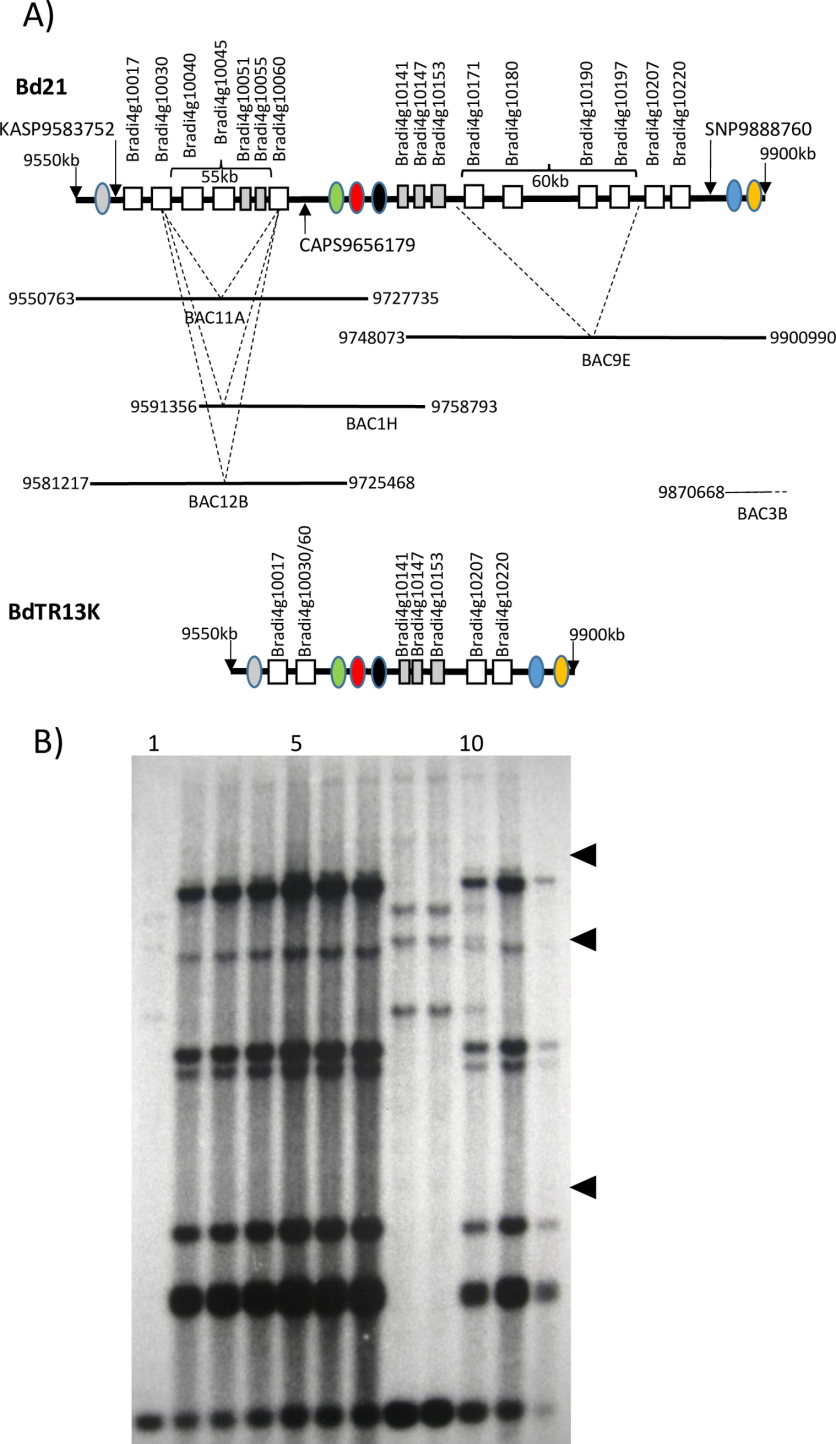
Structure of the *Yrr2* locus. A) Diagram depicting the *Yrr2* locus. NLR genes present at the Bd21 *yrr2* locus are shown as white boxes labelled with gene accession numbers (numerous other genes at this locus are not shown). Beneath are the equivalent regions of sequenced BAC clones from BdTR13k with equivalent nucleotide co-ordinates of the Bd21 chromosome 4 sequence shown. NLR genes absent in these BACs are shown with dashed lines. Deleted regions are marked on the upper Bd21 sequence in parentheses with deletion sizes indicated. The structure of the BdTR13k *Yrr2* locus is shown beneath. Coloured ovals represent nonNLR genes described in Fig 8. Grey boxes represent truncated NLR genes. B) DNA blot analysis of *B*. *distachyon* BdTR13k x BdTR10h F4 DNAs. DNAs were restricted with *HindIII* and hybridised with a probe that encoded a 387 bp sequence derived from NLR genes at the *Yrr2* locus. DNAs from parental lines BdTR13k (*Yrr2* haplotype) and Bd21 (*yrr2* haplotype) are shown in lanes 1 and 12, respectively. The remaining lanes contain DNAs from F4 progeny of these lines which segregate for the *Yrr2* locus. Band intensities reflect DNA loadings. Arrowheads indicate molecular weight mobilities of 8, 5 and 2 kb.

Genotyping of a critical F_3_ recombinant, plant 41 (*yrr1/yrr1; Yrr2*/*Yrr2*), whose progeny all showed *Pst* immunity (20 plants screened with *Pst* and analysed microscopically in three independent experiments) with SNP, KASP and CAPs markers (S7 Table), coupled with plant F_3_ 237 which showed macroscopic lesions, defined the *Yrr2* locus to a 300 kb interval between nucleotides 9,583,752 and 9,888,760 bp of the chromosome 4 reference sequence (Fig 7A). Within this genomic interval in the Bd21 genome 37 genes are annotated (S8 Table), 16 of which encode NLR genes (Fig 7A). Five of these 16 NLR genes encode short truncated proteins and are likely pseudogenes (Fig 7A). The NLR proteins encoded by this gene family vary from 53% to 98% amino acid identity.

A total of five BdTR13k BAC clones were isolated from the *Yrr2* interval that collectively covered the region in its entirety ie. from marker KASP9583752 through to marker SNP9888760 (Fig 7A). These BAC clones were isolated by PCR screening using SNP9888760 primers (BAC9E and 3B) and CAPS9656179 primers (BAC 11A and 12B) (S7 Table), while a nonpolymorphic set of primers (materials and methods—primers 9735685) were used to isolate BAC 1H (Fig 7A). These five BAC clones were sequenced and compared with the Bd21 reference genome sequence. Compared with Bd21 the number of NLR genes at the BdTR13k *Yrr2* locus was considerably reduced, consisting of just seven genes (Fig 7A). Three of these genes (Bradi4g10141, Bradi4g10147, Bradi4g10153) encode truncated NLR proteins like their Bd21 equivalents while Bradi4g10220 encodes a full length protein identical in sequence to that present in Bd21 (Table 2). The remaining three genes (Bradi4g10017, Bradi4g10030/10060, Bradi4g10207) encode probable full length proteins distinct to those found at the Bd21 locus (90–98% amino acid identity–Table 2) making them potential gene candidates for *Yrr2*.

**Table 2:** Proteins encoded by NLR genes present at the *Yrr2* locus of *B*. *distachyon* accession BdTR13k compared with nearest equivalent Bd21 proteins.

Protein	No. of a.a. in BdTR13k	No. of a.a. in Bd21	% identity
Bradi4g10017	882	740	98%
*Bradi4g10030/10060	1015	(Bradi4g10030) 1012	92%
*Bradi4g10030/10060	1015	(Bradi4g10060) 1022	90%
Bradi4g10141	216	216	100%
Bradi4g10147	277	452	100%
Bradi10153	345	487	99%
Bradi4g10207	1015	1014	98%
Bradi4g10220	973	973	100%

*Bradi4g10030/10060 appears to be a chimeric gene formed by recombination between Bradi4g10030 and Bradi4g10060 genes hence its product is compared with both proteins.

The sequences of these 3 candidate genes were compared between Tek-2 (yrr2), Tek-4 (yrr2), BdTR10h (yrr2), Luc1 (yrr2) and ABR6 (Yrr2 –see accompanying paper by Bettgenhaeuser et al.). Proteins encoded by Bradi4g10017 genes were truncated in Tek-2 (314 a.a.), Tek-4 (740a.a.), Bd21 (740 a.a.), BdTR10h (774 a.a.) compared with the 882 amino acid proteins encoded by BdTR13K (Table 2), ABR6 and Luc1, with these latter proteins having 99% identity. No accessions encoded proteins identical to the predicted BdTR13k Bradi4g10030/10060 protein (1015 a.a) with ABR6 (658a.a), BdTR10h (830 a.a.) and Tek-4 (658 a.a.) encoding truncated proteins. While all accessions appeared to encode full length Bradi4g10207 proteins none, including the ABR6 protein, were identical to the BdTR13k protein with the Bd21 protein showing greatest identity (98%) (Table 2). This comparative analysis therefore did not unambiguously resolve an obvious *Yrr2* candidate amongst these three genes. (It should be noted that small regions of some these genes derived from whole genome sequences did not have full coverage and hence these small regions were excluded from identity calculations of all proteins).

Sequence analysis indicated that differences in NLR gene numbers between the Bd21 and BdTR13k loci were due to two insertion/deletion events of 55 and 60 kb, respectively. The boundaries of these two events could be identified by flanking sequence homology. Assuming each event was a deletion in BdTR13k, the first appeared to have arisen by recombination between NLR genes Bradi4g10030 and Bradi4g10060, two genes with 92% ORF identity, to produce a potentially functional chimeric NLR gene encoding the 5’ terminus of the former gene and 3’ of the latter. This deletion was found in three different BAC clones demonstrating that it was not a cloning artefact. The second deletion occurred between conserved regions immediately 5’ of NLR genes Bradi4g10171 and Bradi4g10207. The differential haplotype complexity of the *Yrr2* locus in Bd21 and BdTR13k was confirmed by DNA blot analysis of 54 F_4_ individuals from the BdTR13k x Bd21 family (examples shown in Fig 7B). It cannot be ruled out, however, that both events may be diverged duplications in the Bd21 genome arising from unequal crossing over. Regardless of the mechanism, significant variation has evolved between the BdTR13k and Bd21 *Yrr2* haplotypes.

### Identification of rice and wheat homologues of genes present at *Yrr1* and *Yrr2*

**Fig 8:**
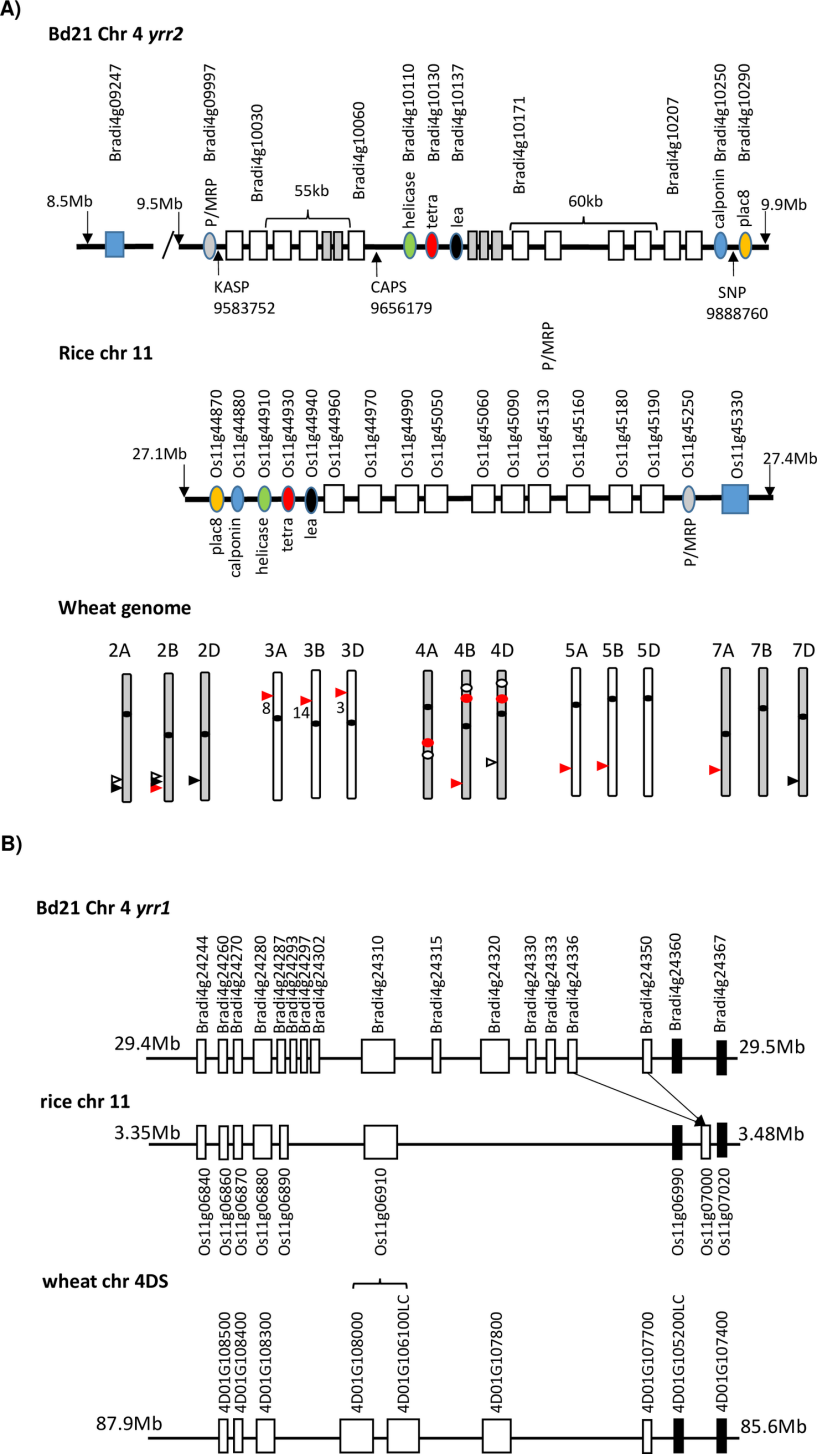
Synteny of *B*. *distachyon Yrr* loci in wheat and rice. A) Structure/location of loci present in *B*. *distachyon*, rice and wheat that encode NLR homologues present at *BdYrr2*. The upper diagram depicts the *B*. *distachyon yrr2* locus present in accession Bd21, with NLR genes shown as white boxes. Six additional nonNLR genes are shown as coloured ovals that are also present at the homologous rice locus located on chromosome 11 (middle diagram), albeit in a different linear order. Rice NLR genes at this locus are shown as white boxes. In the lower diagram the location of sequences in the wheat genome with homology to the *Yrr2* NLR gene Bradi4g10207 are depicted with arrows on the relevant chromosome. Red, black and white arrows indicate high confidence, low confidence and unannotated wheat genes, respectively. Numbered arrows indicate the number of high confidence NLR genes at the wheat locus while unnumbered arrows depict a single gene only. Red dots show loci on wheat chromosome 4 that are regions with some gene co-linearity to non-NLR genes present at the Brachypodium *Yrr2* locus, while white dots on the same wheat chromosomes show the location of potential *Yrr1* homologous loci. Note each diagram depicts relevant genes only and not all genes present at these loci are shown. B) Structure/location of loci present in rice and wheat that show some sequence similarity to the Bd21 *yrr1* locus. Conservation of gene order is shown between the *yrr1* locus of *B*. *distachyon* accession Bd21, a region of rice chromosome 11 and a region of wheat chromosome 4DS. Genes are depicted as boxes with homologous genes aligned vertically. Only rice and wheat genes with homologues in the *B*. *distachyon* genome are illustrated. Arrows show a loss of one of two related genes in the rice genome compared with *Bdyrr1* and a change in gene order. The black boxes indicate *Brachpodium* genes that lie immediately outside of the *yrr1* locus. All wheat genes begin with the prefix (TraesCS). Genes TraesCS4D01G106100LC and TraesCS4D01G108000 are a duplication of the Bradi4g24310 homologue. Note the wheat locus is inverted relative to the Brachypodium and rice locus with nucleotide co-ordinates indicated in Mb.

Homologues of the *Yrr2* locus are present in both the rice and wheat genomes. In rice this NLR gene family consists of 10 tandemly duplicated genes located on chromosome 11 that encode proteins with approximately 60% identity to protein homologues encoded by the *B*. *distachyon Yrr2* (*BdYrr2*) locus (Fig 8A). To further investigate the syntenic relationship of this locus in rice and *B*. *distachyon* additional nonNLR genes in the region were also examined. Six nonNLR genes were identified at *BdYrr2* that encode proteins with substantial homology (65–85% identity) to proteins encoded by the equivalent rice locus, although the relative order of these genes has diverged between these two species (Fig 8A). For both species numerous other genes were also present at the locus for which no reciprocal homologue was present at the locus of the other species. In addition, a NLR gene (Os11g45330) unrelated to the rice *Yrr2* NLR family was located at the locus while the equivalent *B*. *distachyon* gene (Bradi4g09247) was located approximately 1Mb distal to the locus. Previous comparisons between the *B*. *distachyon* and rice genomes have shown that chromosome 4 of *B*. *distachyon* has substantial similarity in gene order and content to rice chromosomes 11, 12 and 4 [43].

Thirty genes with high confidence annotation are present in the Chinese Spring wheat genome that encode proteins with approximately 60% identity to the BdTR13k NLR homologue of Bradi4g10207 (Fig 8A). These are located on chromosomes 2B (1 gene), 3A (8), 3B (14), 3D (3), 4B (1), 5A (1), 5B (1) and 7A (1) (Fig 8A). Nineteen of these 30 genes, present on chromosomes 2B (1), 3A (6), 3B (7), 3D (2), 5A (1), 5B (1) and 7A (1), encode predicted full length proteins. The remaining genes encode either truncated proteins or pseudogenes, although these former proteins could potentially play a role in disease resistance [44]. Additional low confidence and unannotated homologues are also present on these chromosomes (Fig 8A).

The larger NLR gene families present on wheat chromosomes 3AS, 3BS and 3DS are located at the 41, 52 and 30 Mb regions of these chromosomes, respectively, suggesting that they are homoeologous loci. Each locus encompasses 1 (3AS), 1.6 (3BS) and 0.25 Mbs (3DS) of sequence, respectively. Interestingly the non-NLR genes common to the *B*. *distachyon* and rice loci are not located at these wheat 3S loci. Wheat genomic analyses have identified little gene collinearity existing between wheat chromosome 3 and either *B*. *distachyon* chromosome 4 or rice chromosome 11 [45, 46, 47]. It is noteworthy that this wheat chromosome shows less conservation of gene collinearity when compared with other grass chromosomes [47]. Similar potential homoelogous wheat NLR loci are present on wheat chromosome 2L, albeit encoding low confidence and unannotated genes, while related loci are present on two out of three homoleogous chromosomes of 5L and 7L (Fig 8A). Related sequences are also located on chromosome 4B and 4D at 673 and 467 Mb, respectively (Fig 8A).

A number of race specific and adult plant wheat stripe rust resistance loci have been mapped to wheat chromosomes 3BS (eg. *Yr4/YrRub* [48] *Yr30* [49] *Yr57* [50], *Yrns-B1* [55]), 3DS (*Yr49* [49], *Yr66* [49]) and 3AS (*Yr76*)[52]. Comparative genomics was used to locate these wheat *Yrr2* NLR homologues (*TaYrr2*) on chromosome 3S relative to these known wheat *Pst* resistance genes (Table 3). Although there are numerous stripe rust resistance genes on wheat chromosomes 3S none could be definitively located at the *TaYrr2* loci (Table 3).

**Table 3:** Proximity of wheat stripe rust resistance genes to NLR loci on wheat chromosome 3 that are homologous to the Brachypodium *Yrr2* locus.

*Yr* gene	Chr	*Yr* gene location relative to molecular marker	Relationship to *TaYrr2*	Ref
*Yr4/YrRub*	3BS	5cM distal to *Barc75*	*Barc75* is 10 Mb distal to *TaYrr2-3BS*	[48]
*Yr57*	3BS	Between *BS00062676* and *gwm389*	Markers are distal to *TaYrr2-3BS*	[50]
*Yr30*	3BS	Distal to *Xgwm493*	*Xgwm493* is 40 Mb distal to *TaYrr2-3BS*	[49], [51], [53], [54]
*QYr*.*ucw-3BS QTL*	3BS	Distal to *Xgwm493*	*Xgwm493* is 40 Mb distal to *TaYrr2-3BS*	[51], [53], [54]
*Yrns-B1*	3BS	2.5cM proximal to *Xgwm493*	*Yrns-B1* is an APR gene making *TaYrr2-3BS* an unlikely candidate.	[55]
*Yr49*	3DS	1cM from *gwm161*	*gwm161* is located at 7 Mb while *TaYrr2-3DS* is located at 60 Mb	[49] [56]
*Yr66*	3DS	distal to *Yr49* and 2.9 cM from marker *IWB18087*	*TaYrr2-3DS* is proximal to Yr49 and located at 60 Mb region	[49] [57]
*Yr76*	3AS	distal to wmc532 with this marker at 22Mb	*TaYrr2-3AS* is proximal to wmc532 and *Yr76* and is located at 41Mb	[52]

Of further interest is a region on wheat chromosome 4 that has some similar gene co-linearity to the non-NLR genes present at the Brachypodium *Yrr2* locus (S6 Fig). At each wheat chromosome 4 locus non-NLR genes with homology to those found at the Brachypodium *Yrr1* locus are present in the same gene order apart from one large inversion event. In contrast, those regions that encode NLR genes at the Brachypodium *Yrr2* locus have been replaced at these wheat loci with additional unrelated sequence ranging from 70 kb to 3.6 Mb in length. The NLR sequences in the wheat genome that are homologous to those present at the Brachypodium *Yrr2* locus have therefore undergone significant expansion and relocation since the divergence of wheat and Brachypodium.

A similar sequence to the Bd21 *yrr1* locus is also located on rice chromosome 11 (Fig 8B). Homologues of eight genes from the Bd21 *yrr1* locus are present in a similar collinear gene order in the rice genome, although seven *B*. *distachyon* gene sequences are absent in this rice interval (Fig 8B). Numerous other genes are also present at both the rice and Brachypodium loci for which no homologue is present at the locus of the reciprocal species. Potential regions of synteny with *Bdyrr1*are also present on wheat chromosome 4 as illustrated for the 86 Mb region of chromosome 4DS (Fig 8B), while similar genes are also present on chromosomes 4AL (500Mb) and 4BS (123 Mb) (S7 Fig; Fig 8A). A number of wheat stripe rust resistance genes are located on wheat chromosomes 4DS and 4AL, however, none are closely associated with *TaYrr1-DS* or *TaYrr1-4AL* (Table 4). No stripe rust resistance genes have been reported in proximity of the *TaYrr1-4BS* locus.

**Table 4:** Wheat stripe rust resistance genes on wheat chromosome 4 and their proximity to a region with homology to the Brachypodium *Yrr1* locus.

*Yr* gene	Chr	Close Marker	Relationship to *TaYrr1*	Reference
*Yr28*	4DS	between marker *Xbcd265*-*Xwgm634*	*Xwgm634* is located at 32.3Mb while *TaYrr1-4DS* is located at 86 MB	[58]
*Yr51*	4AL	distal to marker *sun154*	*sun154* (derived from wheat EST Genbank BE444404) is located at 715Mb while *TaYrr1-4AL* is at 500 Mb	[59]
*Yr60*	4AL	distal to marker *sun154*	*sun154* (derived from wheat EST Genbank BE444404) is located at 715Mb while *TaYrr1-4AL* is at 500 Mb	[60]

## Discussion

This study demonstrates that some components of resistance to *Pst* in *B*. *distachyon* are under simple genetic control with two dominant loci, *Yrr1* and *Yrr2*, identified that confer highly restricted pathogen growth, albeit on an underlying background of incompatibility. The *B*. *distachyon* accessions that allowed the most *Pst* colonisation in this study, however, are still obviously resistant indicating that genes additional to *Yrr1* and *Yrr2* underlie *Pst* resistance. The observation that the *Yrr2* locus is both race specific and encodes a polymorphic NLR gene family suggests that a resistance mechanism typical of host resistance is likely to be involved in *B*. *distachyon* resistance to *Pst*.

*B*. *distachyon* is more closely related to wheat than is rice, which may account for the more apparent phenotypic variation in resistance to *Pst*, a nonadapted pathogen of this model grass species [10, 11]. Paradoxically, however, the rice genome shows greater similarity to *BdYrr2* than does the wheat genome. In wheat, NLR homologues of the *BdYrr2* locus have expanded to other chromosomal locations while a residual progenitor of the *Yrr2* locus that encodes nonNLR genes exists on chromosome 4. In contrast, rice maintains an NLR cluster that is combined with nonNLR genes also present at the *BdYrr2* locus. Unlike the extensive rearrangement of *Yrr2* NLR homologues in the wheat genome both rice and wheat maintain similar levels of gene collinearity with the *BdYrr1* locus.

While phenotypic variation within a species to nonadpated pathogen infection is often under polygenic control [24, 25, 26, 27, 28, 29] exceptions, in addition to this *B*. *distachyon* study, have been reported. In wheat, resistance to *P*. *coronata* f. sp. *hordei* [61] and *P*. *striiformis* f. sp. *hordei* [62] was conferred by a single dominant gene in each case. Similarly monogenic resistance to *Pst* has been reported in barley [63, 64, 65]. It is noteworthy that these latter examples involve closely related host and nonhost species and pathogen *formae speciales*.

Resistance to nonadapted pathogens is generally considered to be durable as pathogen colonisation of new plant species is rare on short term evolutionary time frames [5, 6]. The results reported here, however, indicate that not all components of this resistance are likely to be durable. The *B*. *distachyon Yrr2* gene shows *Pst* race specificity, only being effective against some *Pst* isolates. The resistance provided by this locus, if functionally transferred to a host species, is therefore likely to only be transiently effective against avirulent *Pst* races given virulent pathogen races already exist.

Two alternative hypotheses may explain the race specificity of the *Yrr2* gene. The geographical co-localisation of *B*. *distachyon* with wild wheat and its relatives suggest these species may have a close co-speciation history [66, 67]. If so *B*. *distachyon* may have had a long exposure to *P*. *striiformis* from inoculum provided by adjacent *Pst* susceptible wheat plants. The ability of some *Pst* isolates to grow on *yrr2 B*. *distachyon* accessions and very occasionally sporulate may represent the early initiation of a host species jump of this pathogen.

An alternative hypothesis is that it is unlikely that *Pst* has directly evolved to overcome the *B*. *distachyon Yrr2* gene as sporulation rarely occurs on this species. More likely is that *Yrr2* recognises an effector or effector modification that is also recognised by a resistance gene present in a host of *Pst*, thereby providing a clear selective advantage for loss or mutation of this *Pst* effector in the host pathosystem. However, we could not identify a described wheat stripe rust resistance that co-locates with the major NLR gene clusters on wheat chromosome 4 with homology to *BdYrr2* NLR sequences. A question of interest is whether the *B*. *distachyon Yrr2* gene exists solely to provide resistance against *Pst* and potentially other nonadapted pathogens, or if it also recognises adapted *B*. *distachyon* pathogens. Given that singularly this gene appears to provide little selective advantage against *Pst* infection it seems likely that it may also provide resistance against adapted *B*. *distachyon* pathogen species.

This possibility raises some interesting implications. Some components of resistance to nonadapted pathogens that overlap with host resistance (eg. potential recognition of a common effector molecule or activity) may be profoundly affected by host/pathogen dynamics. In the nonhost, a single resistance gene superimposed on an underlying polygenic background of additional resistance is unlikely to result in nonadapted pathogen evolution as the pathogen rarely sporulates. In contrast, it is well established that a host resistance gene that recognises a pathogen effector will exert significant selective pressure for pathogen effector loss or alteration as the end result is extensive pathogen reproduction. If the same effector is also recognised by a gene in a nonhost plant, where it contributes to resistance to the nonadapted pathogen, this latter gene will simultaneously be overcome as a consequence of coevolution between the host and pathogen. Effector based resistance that is common with host resistance is therefore unlikely to remain durable in the nonhost species.

However, it is possible that some NLRs present in nonhost species have evolved to recognise effectors or effector activities in nonadated pathogen species that are essential and not readily modified by mutation. The enforced conservation of these molecules would result in ubiquitous recognition of a pathogen species (ie. broad spectrum). In an accompanying paper Bettgenhaeuser and colleagues (2018) describe the identification of a third *B*. *distachyon Pst* resistance locus, *Yrr3*, that is also possibly conferred by an NLR gene. However, this gene recognises all isolates of *Pst* tested in addition to *P*. *striiformis* f sp. *hordei* the causative pathogen of barley stripe rust disease.

Genetic haplotype analysis has reduced the *Yrr1* locus from 15 candidates to five genes that are polymorphic between immune and more susceptible accessions (Table 1). For all 15 genes at the locus, RNAseq analysis of uninfected tissue in the accompanying paper by Bettgenhaeuser et al shows no consistent transcriptional differences between ABR6 (Yrr1) and yrr1 lines that could account for resistance. A transcriptional difference existing between nonpolymorphic genes therefore appears unlikely to confer immunity, although differential pathogen induction of gene expression cannot be excluded.

In contrast to *Yrr2*, no NLR genes are present at the *Yrr1* locus suggesting a different resistance mechanism. However, it is noteworthy that the resistance phenotypes of *Yrr1* and *Yrr2* are very similar both macroscopically and microscopically. In both instances cell death at infection sites is limited, more so in BdTR13k than BdTR10h. Cell death, while commonly associated with NLR protein signalling, is not always apparent [68]. The separation of browning at lesions and restricted *Pst* growth in the BdTR10h x Tek-4 family [41] demonstrates that additional genes that are not essential for resistance also contribute to infection phenotypes of nonadapted pathogens.

Our previous studies indicate that neither *Yrr1* nor *Yrr2* appear to function against other nonadapted cereal rust pathogens; specifically *P*. *graminis* f. sp. *tritici*, the causal agent of wheat stem rust disease, *P*. *graminis* f. sp. *avenae* (oat stem rust pathogen) and *P*. *graminis* f. sp. *phalaridi* (phalaris rust pathogen) [41]. When compared with other *B*. *distachyon* accessions BdTR13k which contains both *Yrr1* and *Yrr2* did not further restrict the growth of these cereal pathogens and was colonised with larger infection sites and small sporulating pustules. However, it can’t be ruled out that these *Yrr* genes may provide resistance to other *P*. *graminis* isolates not tested in these studies.

Germplasm screening of natural accessions for relatively subtle differential infection outcomes of nonadapted pathogens often identifies an underlying complex genetic inheritance [24, 25, 26, 27, 28, 29]. Fine mapping and ultimately cloning of the genes underlying polygenic resistance mechanisms is a difficult proposition due to the often minor effects conferred by each locus coupled with subtle phenotype variation. Here, *B*. *distachyon* is an exception to this generalisation with two independent, dominant loci each conferring readily discernible phenotypes. The *Yrr1* locus has been refined to five candidate genes that encode a glyoxal oxidase, a sentrin/SUMO protease, a CBS-domain protein and two proteins of unknown function, respectively, while the race-specific *Yrr2* locus has been reduced to three candidate NLR genes. Future studies will delineate which specific gene(s) at each locus is responsible for macroscopic immunity by transforming *B*. *distachyon* with these candidate genes.

Of great interest will be to test the ability of these genes to provide *Pst* resistance in wheat, a host of this pathogen. Previous studies have demonstrated that in some instances genes that provide resistance to nonadapted pathogens in one species can be transferred to host species to provide resistance to virulent races of the same pathogen. Examples include the transfer of the *EF-TU (ERF)* PAMP receptor *(ERF)* from Arabidopsis to Solanaceous species [69] the maize *Rxo1* NLR gene transferred to rice [70] and Arabidopsis resistance genes to Asian soybean rust (*Phakopsora pachyrhizi*) transferred into soybean [71]. These observations demonstrate that genes providing resistance against nonadapted pathogens can be a valuable source of disease resistance for crop hosts of these diseases if they can be isolated amongst an often complex background of genetic and mechanistic redundancy.

## Materials and methods

### Plant growth and rust propagation

*B*. *distachyon* plants were grown in a soil/compost (1:1) mixture at 18 ^o^C under standard greenhouse growth conditions or alternatively in growth cabinets at 18 ^o^C with a 16 hour photoperiod. *Pst* isolates were propagated on wheat cultivar Morocco and urediniospores harvested by shaking infected plants over aluminium foil. For *Pst* infection *B*. *distachyon* plants were inoculated with freshly harvested urediniospores as an aerosol spray, lightly misted with water and then incubated at 10 ^o^C overnight before being returned to 18 ^o^C constant growth conditions. Rust symptoms were scored after approximately 3 weeks. Four *Pst* pathotypes were used for infection assays; 110 E143 A+ [Plant Breeding Institute accession number (AN) 861725], 134 E16 A+ [AN 021510], 104 E137 A–[AN 821559] and 108 E141 A- [AN 832002].

### Molecular biological analyses

DNA extractions, PCR analysis and DNA blot analyses were undertaken as previously described [72]. A 387 bp fragment encoding a region of the NLR genes present at the *Yrr2* locus was amplified using primer 5’-TATTGAGAAGATCTTTGAGCA-3’ and primer 5’-TCCCCTTCCATAGATGCTGCC-3’ and used as a probe for DNA hybridisation.

### DNA sequencing and bioinformatics

DNA sequencing of genomic DNA pools was undertaken by the Australian Genome Research Facility Ltd using Illumina sequencing with 100 bp paired end reads. DNA sequences were aligned to the Bd21 reference genome and SNPs and indels called using PARTEK and CASAVA programs. The Bd21 reference genome sequence is located at PlantGDB (http://www.plantgdb.org) and the Joint Genome Institute Genome Portal (http://genome.jgi.doe.gov/). Rice genome sequence comparisons were made using the rice genome database OsGDB. Wheat genomic sequences were obtained from the International Wheat Genetics Sequencing Consortium (IWGSC) data repository at URGI-INRA (https://wheat-urgi.versailles.inra.fr/Seq-Repository/Assemblies) and NCBI wheat chromosome 3B nucleotide sequence (Genbank HG670306).

### Whole genome shotgun sequencing of BdTR10h and BdTR13k

Genomic DNA of BdTR10h and BdTR13k was extracted using a standard CTAB-based extraction protocol. TruSeq libraries were generated from gDNA and mean insert sizes were 840 bp for BdTR10h and 993 bp for BdTR13k. Library preparation and sequencing was performed at the Earlham Institute (previously known as The Genome Analysis Centre, Norwich, UK). Sequencing was carried out using 100 bp paired-end reads on an Illumina HiSeq 2500 sequencer and yielded 71 and 61 million raw reads for BdTR10h and BdTR13k, respectively.

### Haplotype and phylogenetic analysis

Resequencing data for several *B*. *distachyon* accessions were obtained from the Joint Genome Institute Genome Portal (http://genome.jgi.doe.gov/) (S9 Table). These sequence data were produced by the US Department of Energy Joint Genome Institute (http://www.jgi.doe.gov/) in collaboration with the user community. Illumina reads were quality controlled using Trimmomatic (Version 0.33) with the following parameters: ILLUMINACLIP:TruSeq3-PE.fa:2:30:10 LEADING:5 TRAILING:5 SLIDINGWINDOW:4:15 MINLEN:36. Alignments to the Bd21 reference (v3.0) were performed with bwa mem (version 0.7.5a-r405) with default parameters. Samtools (version 0.1.19-96b5f2294a) was used to convert sam into bam files (samtools view) with the requirement that reads mapped in a proper pair (-f2), sort the bam file (samtools sort), remove duplicate reads (samtools rmdup), and generate an mpileup file (samtools mpileup). Coverage of reads was determined using bedtools (version v2.17.0; bedtools genomecov -d -split). SNPs and InDels were called using VarScan (version 2.3.8) with default parameters.

The QKgenome suite (version 1.1.2) of Python scripts were used to assess the haplotype diversity within genes at the *Yrr1* locus in several *B*. *distachyon* accessions. QKgenome_conversion.py was used to evaluate nucleotide variation with the requirement of a read depth of 20 and masking of sequence under this threshold (-m command). Only the first gene model for each gene was used. SNPs and InDels were called based on a frequency threshold of 90%. All genes with InDels that disrupted the coding sequence and mutations with early stop codons were not included in the analysis. A multiple sequence alignment of polymorphic sites was generated using QKgenome_phylogeny.py. The phylogenetic tree was constructed with RAxML (version 8.2.9) using the GTRCAT nucleotide model and rapid hill-climbing mode. Bootstrap support was determined with 2,000 bootstraps, which were found to be sufficient based on the bootstrap convergence test (command -I autoMRE). The neighbour joining tree was generated using Phylip (version 3.695) using default parameters. Bootstrap support was performed with 1,000 bootstraps.

### Accession numbers

Sequence raw reads and assemblies for BdTR10h and BdTR13k were deposited in NCBI under BioProject PRJNA377287. Individual gDNA whole genome sequencing reads include accession numbers SRR5298268 (BdTR10h) and SRR5298269 (BdTR13k). The QKgenome suite of Python scripts described in this manuscript have been deposited on GitHub (https://github.com/matthewmoscou/QKgenome).

### Microscopy

Microscopy of fungal infection structures was undertaken as previously described [22, 41]. Briefly, harvested leaf samples were autoclaved in 1M KOH and then neutralised in a 50 mM Tris pH 7.5 solution. Samples were then stained with a 20 μg/ml solution of wheat germ agglutinin conjugated to fluorescein isothiocyanate and visualised under blue light. The same tissues were examined for autofluorescence using UV light.

### Marker analysis

PCR products were amplified using a Phire Plant Direct PCR Kit (Thermo scientific, USA). For sequencing based markers, PCR products were purified for sequencing using alkaline phosphatase and exonuclease I [73] and sequenced using primer P1. For CAPS marker 9.656179, PCR product was digested with *NarI* (New England Biolabs, USA) and fragments were resolved on a 1% agarose gel. For KASP marker 9.583752, competitive allele specific polymerase chain reaction was performed (KASP; LGC genomics, UK).

### BAC clone library production and screening

*B*. *distachyon* BAC libraries for accession BdTR13k, Tek-4 and BdTR10h were produced by Bio S and T (Quebec, Canada). Individual BACs were isolated by PCR screening of BAC clone pools undertaken by Bio S and T. BAC DNA was purified using an Epicenter BACMAX DNA purification kit and clones then deep sequenced at Kansas State University using Illumina MiSeq sequencing. BAC1H was isolated using PCR primers 9735685F (TTGCTGAGCTTCAAGTGGTG) and 9735685R (ATTCCATTGATGACCGCAGC).

## Supporting information

S1 Fig Phenotypic segregation of *Yrr1* amongst BdTR10h x Tek-4 progeny. (PPTX)

S2 Fig *Pst* analysis of F_3_ families from critical recombinant F_2_ plants. (PPTX)

S3 Fig Neighbor joining phylogenetic tree showing two distinct haplotypes exist at the *Yrr1* locus. (PPTX)

S4 Fig Comparison of Bradi4g24315 proteins. (PPTX)

S5 Fig *Pst* infection phenotypes and segregation ratios of F_5_ families from F_4_ plants 93 and 56. (PPTX)

S6 Fig Region of synteny on wheat chromosome 4 that maintains some gene collinearity with non-NLR genes present at the Brachypodium *Yrr2* locus. (PPTX)

S7 Fig Location of co-linear wheat sequences with homology to Brachypodium genes present at the *Yrr1* locus. (PPTX)

S1 Table PCR sequencing markers at the *Yrr1* locus. (DOCX)

S2 Table Genes annotated in the Brachypodium v3.1 genome that are present at the *Yrr1* locus. (XLSX)

S3 Table SNPs present in 11 genes at the *Yrr1* locus in a panel of Brachypodium accession. (XLSX)

S4 Table Pseudogenes excluded from the *Yrr1* haplotype analysis. (XLSX)

S5 Table *Yrr1* SNP haplotype analysis and *Pst* infection phenotypes. (XLSX)

S6 Table Comparison of Bradi4g24366 between Brachypodium accessions. (XLSX)

S7 Table PCR markers at the *Yrr2* locus. (DOCX)

S8 Table Genes annotated in the Brachypodium v3.1 genome that are present at the *Yrr2* locus. (XLSX)

S9 Table Source of resequencing reads for a diverse panel of *B*. *distachyon* accessions. (DOCX)
