# Supplementary material for: Components of Brachypodium distachyon resistance to nonadapted wheat stripe rust pathogens are simply inherited
Source: PLoS Genet. 2018 Sep 28;14(9):e1007636. doi: 10.1371/journal.pgen.1007636 (PMC6161853; doi:10.1371/journal.pgen.1007636)
Supplement: S1 Fig — (PPTX) [file pgen.1007636.s001.pptx]

## Slide 1
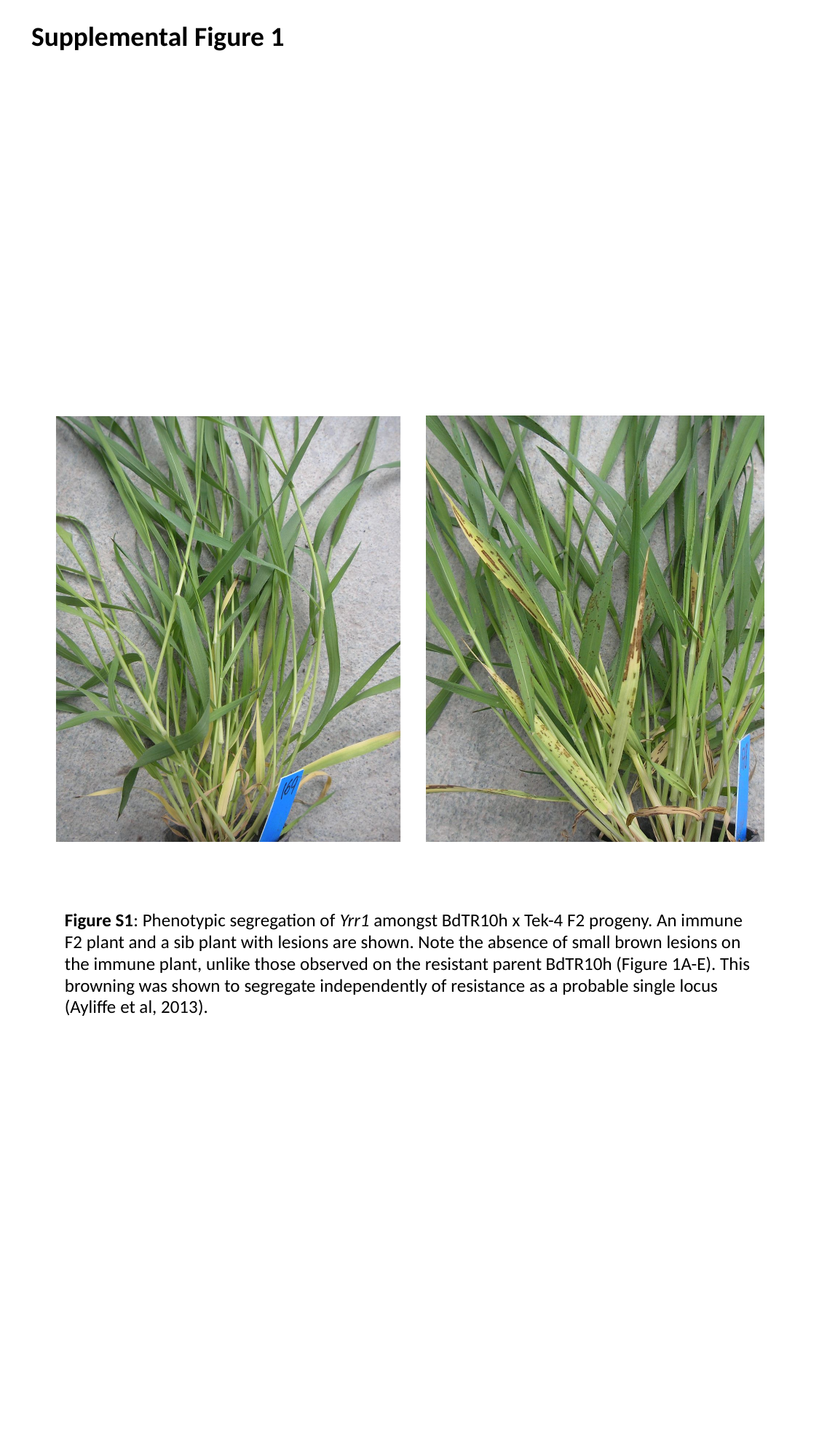

Supplemental Figure 1
Figure S1: Phenotypic segregation of Yrr1 amongst BdTR10h x Tek-4 F2 progeny. An immune F2 plant and a sib plant with lesions are shown. Note the absence of small brown lesions on the immune plant, unlike those observed on the resistant parent BdTR10h (Figure 1A-E). This browning was shown to segregate independently of resistance as a probable single locus (Ayliffe et al, 2013).
