# Supplementary material for: Components of Brachypodium distachyon resistance to nonadapted wheat stripe rust pathogens are simply inherited
Source: PLoS Genet. 2018 Sep 28;14(9):e1007636. doi: 10.1371/journal.pgen.1007636 (PMC6161853; doi:10.1371/journal.pgen.1007636)
Supplement: S2 Fig — (PPTX) [file pgen.1007636.s002.pptx]

## Slide 1
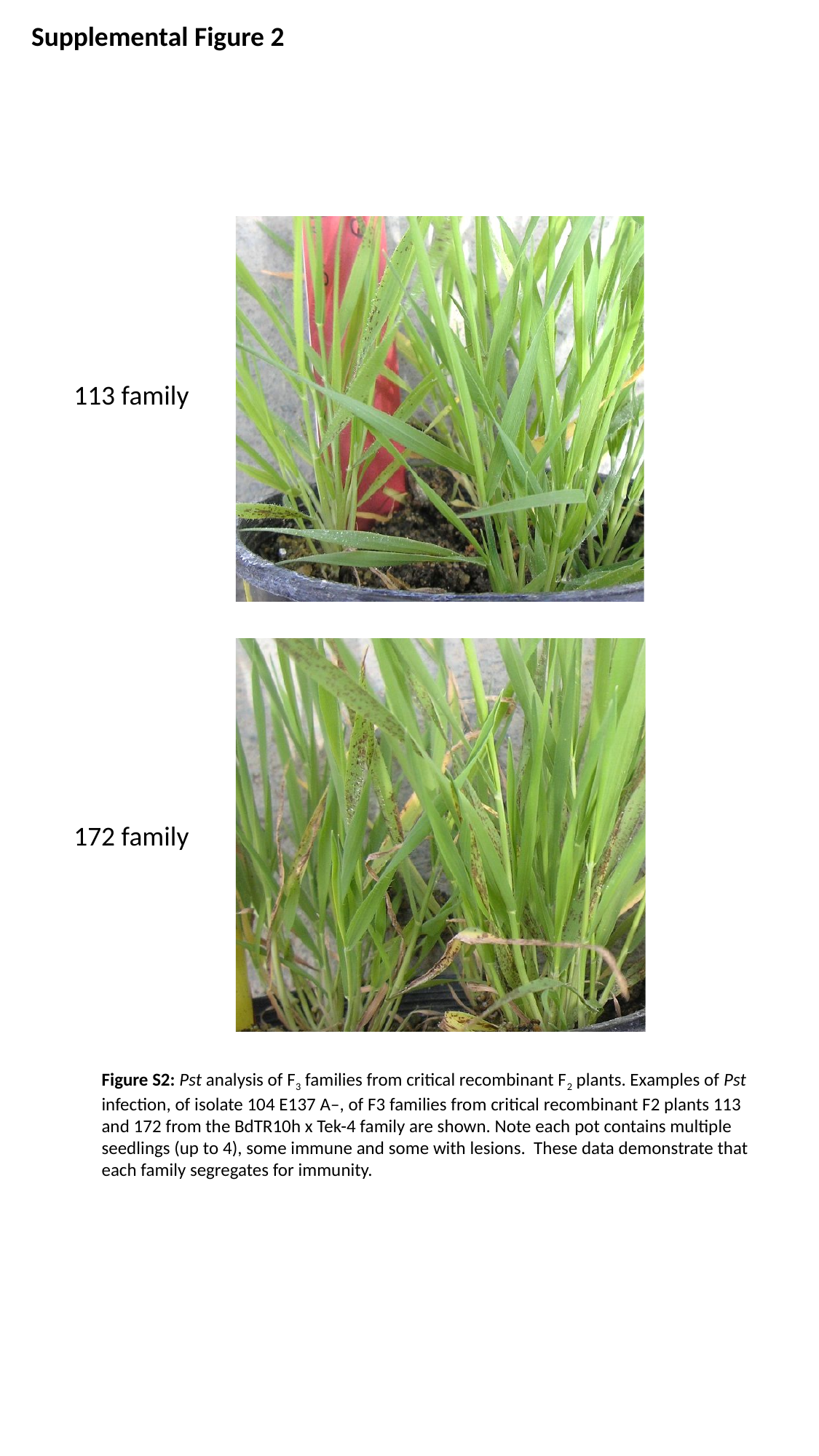

Supplemental Figure 2
113 family
172 family
Figure S2: Pst analysis of F3 families from critical recombinant F2 plants. Examples of Pst infection, of isolate 104 E137 A–, of F3 families from critical recombinant F2 plants 113 and 172 from the BdTR10h x Tek-4 family are shown. Note each pot contains multiple seedlings (up to 4), some immune and some with lesions. These data demonstrate that each family segregates for immunity.
