# Supplementary material for: Components of Brachypodium distachyon resistance to nonadapted wheat stripe rust pathogens are simply inherited
Source: PLoS Genet. 2018 Sep 28;14(9):e1007636. doi: 10.1371/journal.pgen.1007636 (PMC6161853; doi:10.1371/journal.pgen.1007636)
Supplement: S3 Fig — (PPTX) [file pgen.1007636.s003.pptx]

## Slide 1
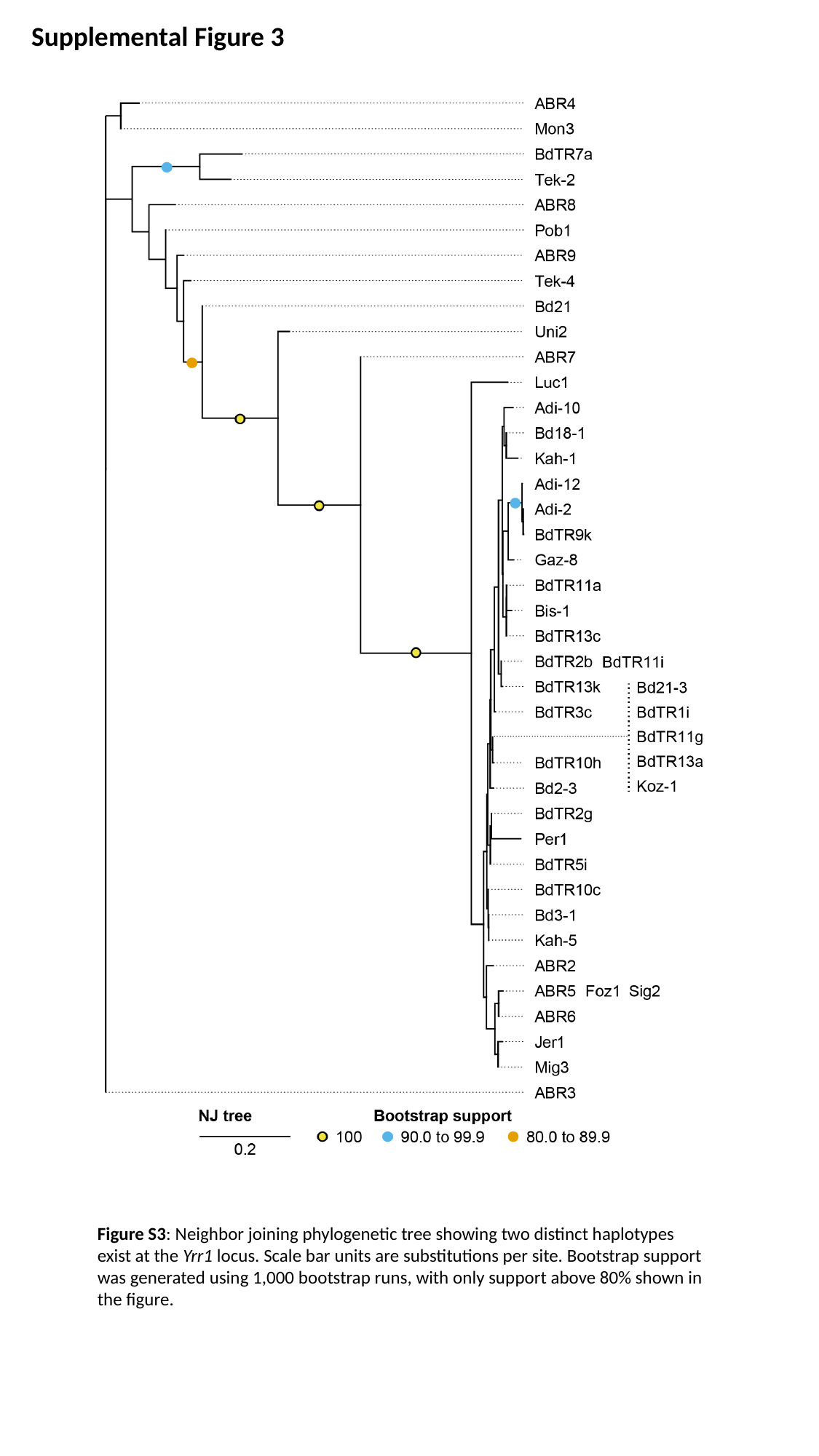

Supplemental Figure 3
Figure S3: Neighbor joining phylogenetic tree showing two distinct haplotypes exist at the Yrr1 locus. Scale bar units are substitutions per site. Bootstrap support was generated using 1,000 bootstrap runs, with only support above 80% shown in the figure.
