# Supplementary material for: Components of Brachypodium distachyon resistance to nonadapted wheat stripe rust pathogens are simply inherited
Source: PLoS Genet. 2018 Sep 28;14(9):e1007636. doi: 10.1371/journal.pgen.1007636 (PMC6161853; doi:10.1371/journal.pgen.1007636)
Supplement: S4 Fig — (PPTX) [file pgen.1007636.s004.pptx]

## Slide 1
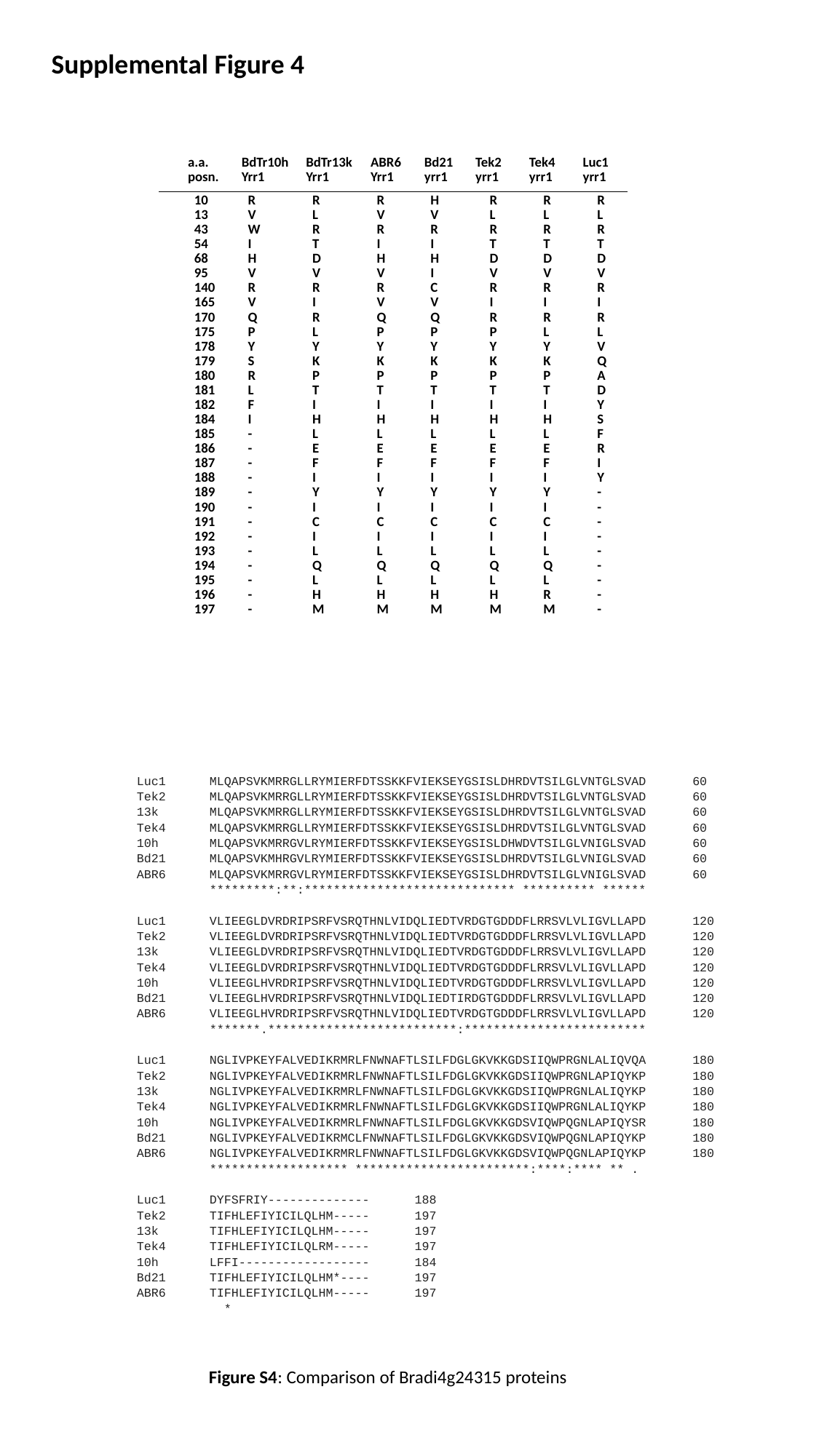

Supplemental Figure 4
| | a.a. posn. | BdTr10h Yrr1 | BdTr13k Yrr1 | ABR6 Yrr1 | Bd21 yrr1 | Tek2 yrr1 | Tek4 yrr1 | Luc1 yrr1 | |
| --- | --- | --- | --- | --- | --- | --- | --- | --- | --- |
| | 10 13 43 54 68 95 140 165 170 175 178 179 180 181 182 184 185 186 187 188 189 190 191 192 193 194 195 196 197 | R V W I H V R V Q P Y S R L F I - - - - - - - - - - - - - | R L R T D V R I R L Y K P T I H L E F I Y I C I L Q L H M | R V R I H V R V Q P Y K P T I H L E F I Y I C I L Q L H M | H V R I H I C V Q P Y K P T I H L E F I Y I C I L Q L H M | R L R T D V R I R P Y K P T I H L E F I Y I C I L Q L H M | R L R T D V R I R L Y K P T I H L E F I Y I C I L Q L R M | R L R T D V R I R L V Q A D Y S F R I Y - - - - - - - - - |
| --- | --- | --- | --- | --- | --- | --- | --- | --- |
Luc1 MLQAPSVKMRRGLLRYMIERFDTSSKKFVIEKSEYGSISLDHRDVTSILGLVNTGLSVAD	60
Tek2 MLQAPSVKMRRGLLRYMIERFDTSSKKFVIEKSEYGSISLDHRDVTSILGLVNTGLSVAD	60
13k MLQAPSVKMRRGLLRYMIERFDTSSKKFVIEKSEYGSISLDHRDVTSILGLVNTGLSVAD	60
Tek4 MLQAPSVKMRRGLLRYMIERFDTSSKKFVIEKSEYGSISLDHRDVTSILGLVNTGLSVAD	60
10h MLQAPSVKMRRGVLRYMIERFDTSSKKFVIEKSEYGSISLDHWDVTSILGLVNIGLSVAD	60
Bd21 MLQAPSVKMHRGVLRYMIERFDTSSKKFVIEKSEYGSISLDHRDVTSILGLVNIGLSVAD	60
ABR6 MLQAPSVKMRRGVLRYMIERFDTSSKKFVIEKSEYGSISLDHRDVTSILGLVNIGLSVAD	60
 *********:**:***************************** ********** ******
Luc1 VLIEEGLDVRDRIPSRFVSRQTHNLVIDQLIEDTVRDGTGDDDFLRRSVLVLIGVLLAPD	120
Tek2 VLIEEGLDVRDRIPSRFVSRQTHNLVIDQLIEDTVRDGTGDDDFLRRSVLVLIGVLLAPD	120
13k VLIEEGLDVRDRIPSRFVSRQTHNLVIDQLIEDTVRDGTGDDDFLRRSVLVLIGVLLAPD	120
Tek4 VLIEEGLDVRDRIPSRFVSRQTHNLVIDQLIEDTVRDGTGDDDFLRRSVLVLIGVLLAPD	120
10h VLIEEGLHVRDRIPSRFVSRQTHNLVIDQLIEDTVRDGTGDDDFLRRSVLVLIGVLLAPD	120
Bd21 VLIEEGLHVRDRIPSRFVSRQTHNLVIDQLIEDTIRDGTGDDDFLRRSVLVLIGVLLAPD	120
ABR6 VLIEEGLHVRDRIPSRFVSRQTHNLVIDQLIEDTVRDGTGDDDFLRRSVLVLIGVLLAPD	120
 *******.**************************:*************************
Luc1 NGLIVPKEYFALVEDIKRMRLFNWNAFTLSILFDGLGKVKKGDSIIQWPRGNLALIQVQA	180
Tek2 NGLIVPKEYFALVEDIKRMRLFNWNAFTLSILFDGLGKVKKGDSIIQWPRGNLAPIQYKP	180
13k NGLIVPKEYFALVEDIKRMRLFNWNAFTLSILFDGLGKVKKGDSIIQWPRGNLALIQYKP	180
Tek4 NGLIVPKEYFALVEDIKRMRLFNWNAFTLSILFDGLGKVKKGDSIIQWPRGNLALIQYKP	180
10h NGLIVPKEYFALVEDIKRMRLFNWNAFTLSILFDGLGKVKKGDSVIQWPQGNLAPIQYSR	180
Bd21 NGLIVPKEYFALVEDIKRMCLFNWNAFTLSILFDGLGKVKKGDSVIQWPQGNLAPIQYKP	180
ABR6 NGLIVPKEYFALVEDIKRMRLFNWNAFTLSILFDGLGKVKKGDSVIQWPQGNLAPIQYKP	180
 ******************* ************************:****:**** ** .
Luc1 DYFSFRIY--------------	188
Tek2 TIFHLEFIYICILQLHM-----	197
13k TIFHLEFIYICILQLHM-----	197
Tek4 TIFHLEFIYICILQLRM-----	197
10h LFFI------------------	184
Bd21 TIFHLEFIYICILQLHM*----	197
ABR6 TIFHLEFIYICILQLHM-----	197
 *
Figure S4: Comparison of Bradi4g24315 proteins
