# Supplementary material for: Components of Brachypodium distachyon resistance to nonadapted wheat stripe rust pathogens are simply inherited
Source: PLoS Genet. 2018 Sep 28;14(9):e1007636. doi: 10.1371/journal.pgen.1007636 (PMC6161853; doi:10.1371/journal.pgen.1007636)
Supplement: S5 Fig — (PPTX) [file pgen.1007636.s005.pptx]

## Slide 1
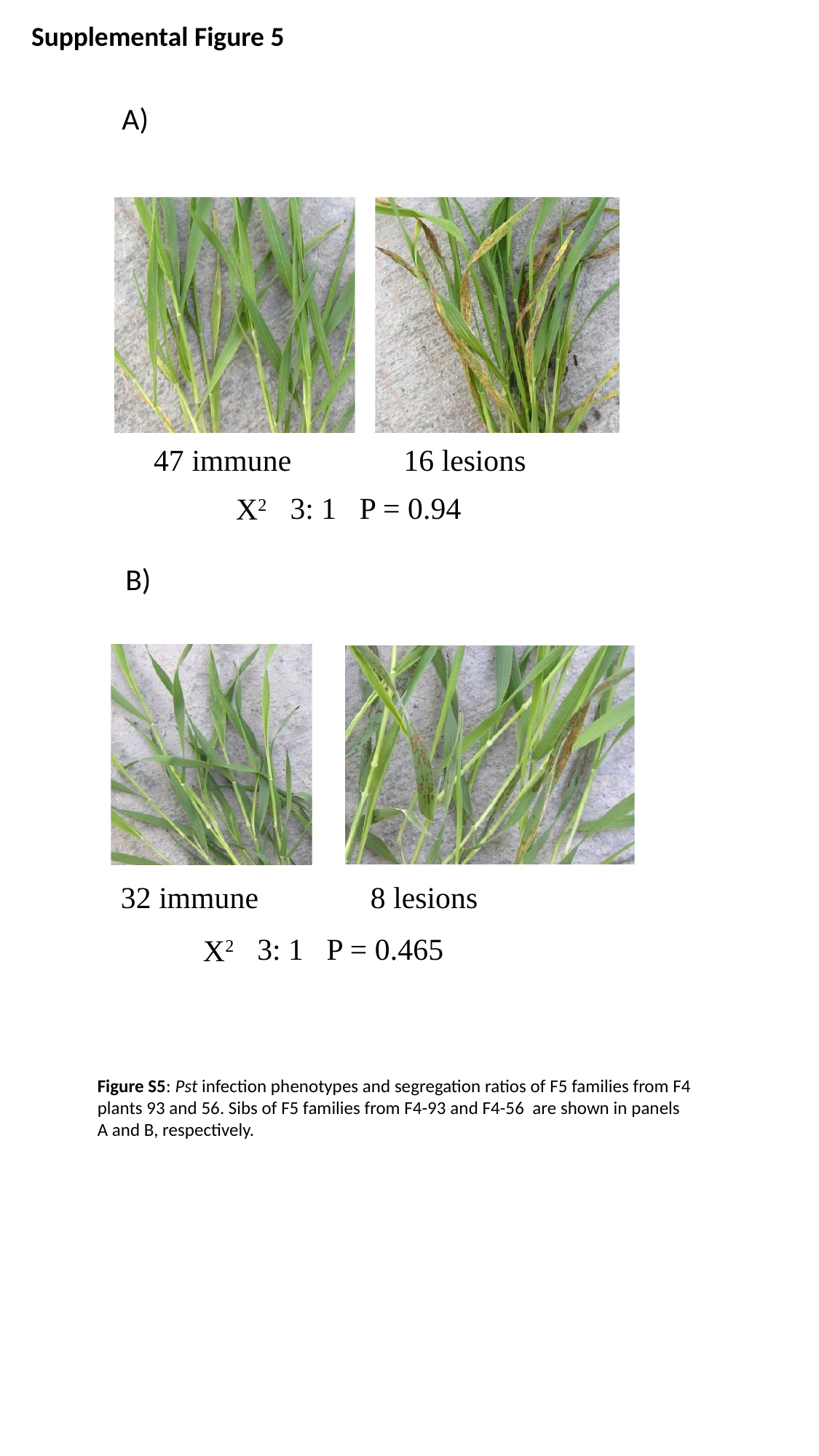

Supplemental Figure 5
A)
47 immune
16 lesions
3: 1 P = 0.94
X2
B)
32 immune
8 lesions
3: 1 P = 0.465
X2
Figure S5: Pst infection phenotypes and segregation ratios of F5 families from F4 plants 93 and 56. Sibs of F5 families from F4-93 and F4-56 are shown in panels A and B, respectively.
