# Supplementary material for: Components of Brachypodium distachyon resistance to nonadapted wheat stripe rust pathogens are simply inherited
Source: PLoS Genet. 2018 Sep 28;14(9):e1007636. doi: 10.1371/journal.pgen.1007636 (PMC6161853; doi:10.1371/journal.pgen.1007636)
Supplement: S6 Fig — (PPTX) [file pgen.1007636.s006.pptx]

## Slide 1
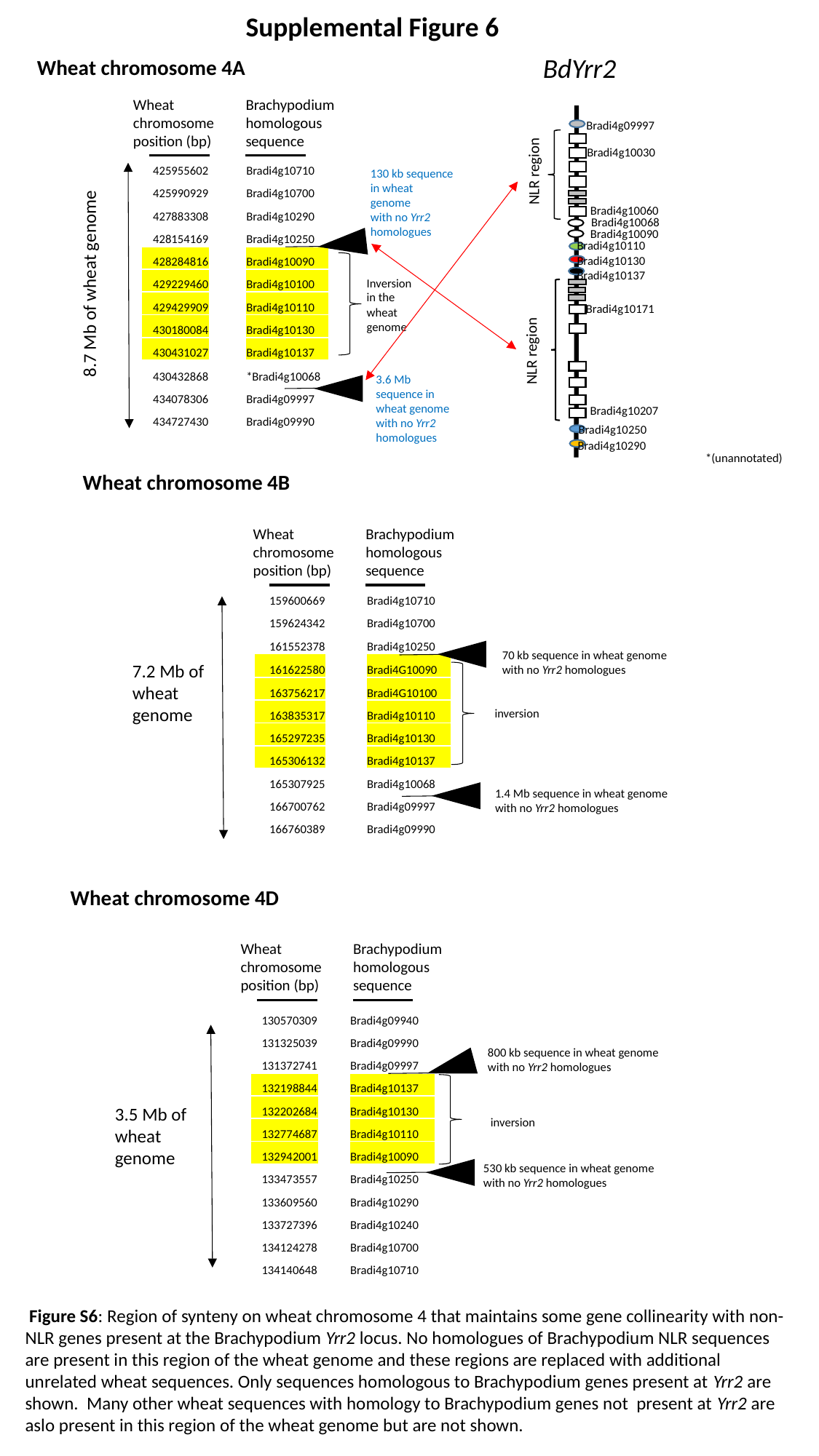

Supplemental Figure 6
BdYrr2
Wheat chromosome 4A
Wheat
chromosome
position (bp)
Brachypodium
homologous
sequence
Bradi4g09997
Bradi4g10030
NLR region
| 425955602 |
| --- |
| 425990929 |
| 427883308 |
| 428154169 |
| 428284816 |
| 429229460 |
| 429429909 |
| 430180084 |
| 430431027 |
| 430432868 |
| 434078306 |
| 434727430 |
| Bradi4g10710 |
| --- |
| Bradi4g10700 |
| Bradi4g10290 |
| Bradi4g10250 |
| Bradi4g10090 |
| Bradi4g10100 |
| Bradi4g10110 |
| Bradi4g10130 |
| Bradi4g10137 |
| \*Bradi4g10068 |
| Bradi4g09997 |
| Bradi4g09990 |
130 kb sequence in wheat genome
with no Yrr2 homologues
Bradi4g10060
Bradi4g10068
Bradi4g10090
Bradi4g10110
Bradi4g10130
8.7 Mb of wheat genome
Bradi4g10137
Inversion in the wheat genome
Bradi4g10171
NLR region
3.6 Mb sequence in wheat genome
with no Yrr2 homologues
Bradi4g10207
Bradi4g10250
Bradi4g10290
*(unannotated)
Wheat chromosome 4B
Wheat
chromosome
position (bp)
Brachypodium
homologous
sequence
| 159600669 |
| --- |
| 159624342 |
| 161552378 |
| 161622580 |
| 163756217 |
| 163835317 |
| 165297235 |
| 165306132 |
| 165307925 |
| 166700762 |
| 166760389 |
| Bradi4g10710 |
| --- |
| Bradi4g10700 |
| Bradi4g10250 |
| Bradi4G10090 |
| Bradi4G10100 |
| Bradi4g10110 |
| Bradi4g10130 |
| Bradi4g10137 |
| Bradi4g10068 |
| Bradi4g09997 |
| Bradi4g09990 |
70 kb sequence in wheat genome
with no Yrr2 homologues
7.2 Mb of
wheat
genome
inversion
1.4 Mb sequence in wheat genome
with no Yrr2 homologues
Wheat chromosome 4D
Wheat
chromosome
position (bp)
Brachypodium
homologous
sequence
| 130570309 |
| --- |
| 131325039 |
| 131372741 |
| 132198844 |
| 132202684 |
| 132774687 |
| 132942001 |
| 133473557 |
| 133609560 |
| 133727396 |
| 134124278 |
| 134140648 |
| Bradi4g09940 |
| --- |
| Bradi4g09990 |
| Bradi4g09997 |
| Bradi4g10137 |
| Bradi4g10130 |
| Bradi4g10110 |
| Bradi4g10090 |
| Bradi4g10250 |
| Bradi4g10290 |
| Bradi4g10240 |
| Bradi4g10700 |
| Bradi4g10710 |
800 kb sequence in wheat genome
with no Yrr2 homologues
3.5 Mb of
wheat
genome
inversion
530 kb sequence in wheat genome
with no Yrr2 homologues
 Figure S6: Region of synteny on wheat chromosome 4 that maintains some gene collinearity with non-NLR genes present at the Brachypodium Yrr2 locus. No homologues of Brachypodium NLR sequences are present in this region of the wheat genome and these regions are replaced with additional unrelated wheat sequences. Only sequences homologous to Brachypodium genes present at Yrr2 are shown. Many other wheat sequences with homology to Brachypodium genes not present at Yrr2 are aslo present in this region of the wheat genome but are not shown.
