# Supplementary material for: Components of Brachypodium distachyon resistance to nonadapted wheat stripe rust pathogens are simply inherited
Source: PLoS Genet. 2018 Sep 28;14(9):e1007636. doi: 10.1371/journal.pgen.1007636 (PMC6161853; doi:10.1371/journal.pgen.1007636)
Supplement: S7 Fig — (PPTX) [file pgen.1007636.s007.pptx]

## Slide 1
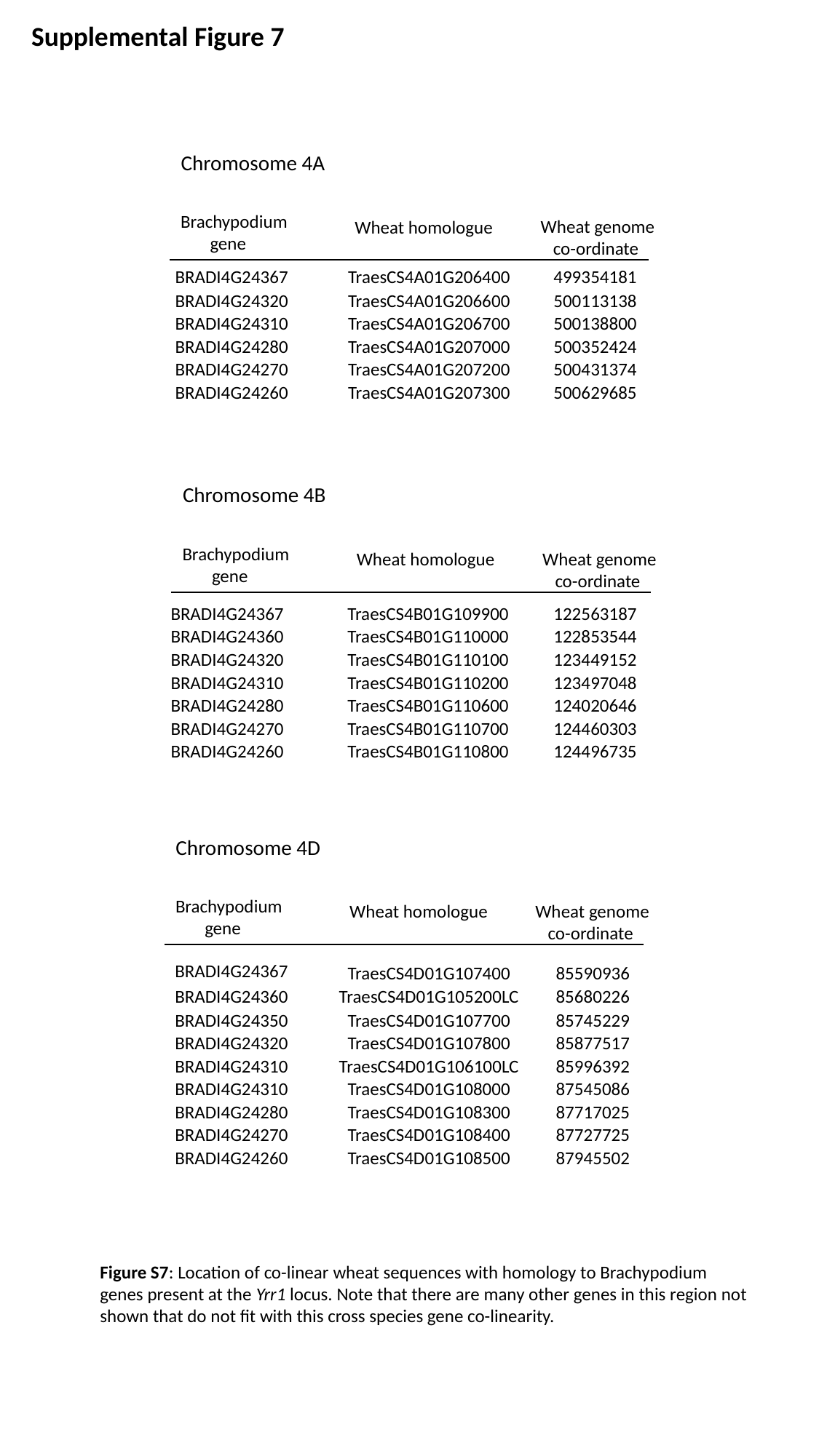

Supplemental Figure 7
Chromosome 4A
Brachypodium
 gene
Wheat homologue
Wheat genome
 co-ordinate
| BRADI4G24367 | TraesCS4A01G206400 | 499354181 |
| --- | --- | --- |
| BRADI4G24320 | TraesCS4A01G206600 | 500113138 |
| BRADI4G24310 | TraesCS4A01G206700 | 500138800 |
| BRADI4G24280 | TraesCS4A01G207000 | 500352424 |
| BRADI4G24270 | TraesCS4A01G207200 | 500431374 |
| BRADI4G24260 | TraesCS4A01G207300 | 500629685 |
Chromosome 4B
Brachypodium
 gene
Wheat homologue
Wheat genome
 co-ordinate
| BRADI4G24367 | TraesCS4B01G109900 | 122563187 |
| --- | --- | --- |
| BRADI4G24360 | TraesCS4B01G110000 | 122853544 |
| BRADI4G24320 | TraesCS4B01G110100 | 123449152 |
| BRADI4G24310 | TraesCS4B01G110200 | 123497048 |
| BRADI4G24280 | TraesCS4B01G110600 | 124020646 |
| BRADI4G24270 | TraesCS4B01G110700 | 124460303 |
| BRADI4G24260 | TraesCS4B01G110800 | 124496735 |
Chromosome 4D
Brachypodium
 gene
Wheat homologue
Wheat genome
 co-ordinate
| BRADI4G24367 | TraesCS4D01G107400 | 85590936 |
| --- | --- | --- |
| BRADI4G24360 | TraesCS4D01G105200LC | 85680226 |
| BRADI4G24350 | TraesCS4D01G107700 | 85745229 |
| BRADI4G24320 | TraesCS4D01G107800 | 85877517 |
| BRADI4G24310 | TraesCS4D01G106100LC | 85996392 |
| BRADI4G24310 | TraesCS4D01G108000 | 87545086 |
| BRADI4G24280 | TraesCS4D01G108300 | 87717025 |
| BRADI4G24270 | TraesCS4D01G108400 | 87727725 |
| BRADI4G24260 | TraesCS4D01G108500 | 87945502 |
Figure S7: Location of co-linear wheat sequences with homology to Brachypodium genes present at the Yrr1 locus. Note that there are many other genes in this region not shown that do not fit with this cross species gene co-linearity.
