# Supplementary material for: Components of Brachypodium distachyon resistance to nonadapted wheat stripe rust pathogens are simply inherited
Source: PLoS Genet. 2018 Sep 28;14(9):e1007636. doi: 10.1371/journal.pgen.1007636 (PMC6161853; doi:10.1371/journal.pgen.1007636)
Supplement: S1 Table — (DOCX) [file pgen.1007636.s008.docx]

**Supplemental Table S1**: PCR sequencing markers at the *Yrr1* locus

| Marker | Primer 1 | Primer 2 |
| --- | --- | --- |
| SNP29017600 | ggcgaattcaagcgtattg | aggtacagtaagtgcttgg |
| SNP29419400 | cccacactcctaaactcg | ccacatggtctgatattctc |
| SNP29421950 | tgagactcaatcacattttagtc | ctcgttccttctttctttacc |
| SNP29426600 | ggtatgtgtttatgatgatatc | gagcaagcagaggaaacctga |
| SNP29428960 | tgggacttcatgttagcaagg | tttgggatgaaaggagtagtc |
| SNP29447000 | ttcgattcgaggtgagcagat | ttgctactctctcatatcagg |
| SNP29472220 | ttcgtacgatgacgagcagtg | aaacgcaacgcaacatcaatt |
| SNP29479980 | aaggtttcacaaggtacatct | ttgctgctgactggctactt |
| SNP29492270 | aagttctaccagccgctttca | gcaagcatacattctttcaac |
| SNP29503850 | catctggaaatattccaggctg | cgaattatatgcagcatatggg |
| SNP29512890 | gtatgaactaggggttttcat | cacatttggagaggtaatgtgc |
| SNP29514990 | aatcctgtccataagctacgt | aagtgatcagcactattcatg |
| SNP29517580 | cttaccagcaacagccacgtc | atacaagaacagaaccacgacg |
| SNP29519000 | taagctcgctgaagtttcgaa | caatgaattggtgaagaatct |
| SNP29530590 | caagttgcgtattacgtatc | gccaactaactatgcaggtt |
| SNP29713400 | cgaggcgtgtttctagggtt | gatgtaacaacaactgtcc |
| SNP29800350 | ttcacacaaagtagaccatatg | gccacgtcactgatccgtgcc |
| SNP30010080 | agaggcgatgcttggagcac | ctagttatgcagtgtctgattc |
| SNP30844060 | ccagtgacgtacggttgcaaag | ttgcagagtatcatgattgcaca |
| SNP30968800 | gagaatggtatgttgagtca | gctgcttgtggctccgagctg |
| SNP31506260 | agactggataatgatggtgg | acagagaagccaagtaacata |
| SNP32015040 | ccctgcacctctactctccaa | gcgtagccgtgaagcataaac |
