# Supplementary material for: Components of Brachypodium distachyon resistance to nonadapted wheat stripe rust pathogens are simply inherited
Source: PLoS Genet. 2018 Sep 28;14(9):e1007636. doi: 10.1371/journal.pgen.1007636 (PMC6161853; doi:10.1371/journal.pgen.1007636)
Supplement: S7 Table — (DOCX) [file pgen.1007636.s014.docx]

**Supplemental** **Table S7**: PCR markers at the *Yrr2* locus

| Marker | Primer 1 | Primer 2 |
| --- | --- | --- |
| SNP6716730 | gagctttaggagtgatgcca | agctggagtattgaagaaga |
| SNP7079570 | agtatgtactgcagattttcc | tagataagcaatggttgatg |
| SNP7550000 | ccacctcacaggactgctaat | ccttggcagggatgaaagcc |
| SNP7733430 | ctcgagggagtataatcatgac | ttcatgtcacagccaaacgtga |
| SNP7761780 | gttgcccttcttcctacacta | taagctgccagaaagctaaac |
| SNP9151000 | ttggattatagcatgatcacc | ccggtgctagccaatgcaat |
| SNP9307760 | gtccttcctgtcagacgcc | gataatctcacgagagtcc |
| SNP9507000 | tggaatatctgttgttcatgg | gatatggagaaggagatattg |
| KASP9583752 | allele 1: gaaggtcggagtcaacggattttgcctaatatcactaggcc  allele 2: gaaggtgaccaagttcatgctttgcctaatatcactaggct | agcttatggtcagctgtctct |
| CAPS9656179 | tattggcacaaaaggtcaactg | atatcagcctcccttagcttcc |
| SNP9714310 | acatgagacagcttagcaac | ttgcccaaccttgcctcatcga |
| SNP9888760 | ttgtggaaggaagtttcac | gcgtatcgaggtcgtcactga |
| SNP9983700 | catcattatgtgatgactctc | aacatactcatctataactgc |
| SNP10358600 | gttatggctgaatagtgtga | cagaagtagcttaaacgga |
| SNP10682160 | aatgataagtttgtgaccca | tctcaaaccattcaccttgg |
| SNP11151340 | taatgtacaagatactgtatg | gcctgttattgtctgtgatgtg |
| SNP11475500 | gccggaattctgggcttgagt | atgctgcttgctagtgacatg |
