# Supplementary material for: Components of Brachypodium distachyon resistance to nonadapted wheat stripe rust pathogens are simply inherited
Source: PLoS Genet. 2018 Sep 28;14(9):e1007636. doi: 10.1371/journal.pgen.1007636 (PMC6161853; doi:10.1371/journal.pgen.1007636)
Supplement: S9 Table — (DOCX) [file pgen.1007636.s016.docx]

**Supplemental Table S9**: Source of resequencing reads for a diverse panel of *B. distachyon* accessions.

| **Accession** | **Source** | **Project** | **File(s)** |
| --- | --- | --- | --- |
| ABR2 | JGI | 1079477 | 2263.4.1841.fastq.gz |
| ABR3 | JGI | 1079478 | 2263.3.1841.fastq.gz |
| ABR4 | JGI | 1079479 | 2300.6.1858.fastq.gz |
| ABR5 | JGI | 1079480 | 2271.6.1846.fastq.gz |
| ABR6 | JGI | 1079481 | 2263.2.1841.fastq.gz |
| ABR7 | JGI | 1079482 | 2288.6.1851.fastq.gz |
| ABR8 | JGI | 1006478 | 6142.6.38445.fastq.gz; 7274.7.65872.TAGCTT.fastq.gz |
| ABR9 | JGI | 1079483 | 2263.6.1841.fastq.gz |
| Adi-10 | JGI | 1078862 | 1702.3.1553.fastq.gz |
| Adi-12 | JGI | 1078870 | 1737.5.1585.fastq.gz |
| Adi-2 | JGI | 1078979 | 2001.4.1706.fastq.gz |
| Bd18-1 | JGI | 1078872 | 1883.6.1652.fastq.gz |
| Bd2-3 | JGI | 1078871 | 1710.3.1556.fastq.gz |
| Bd21 | NCBI | PRJNA32607 | SRR891794_1.fastq.gz; SRR891794_2.fastq.gz; |
|  |  |  | SRR891795_1.fastq.gz; SRR891795_2.fastq.gz |
| Bd21-3 | JGI | 1105691 | 7147.5.62477.ATCACG.fastq.gz |
| Bd3-1 | JGI | 1081435 | 2385.1.1911.fastq.gz; 677.3.845.fastq.gz; 677.4.845.fastq.gz; |
|  |  |  | 677.5.845.fastq.gz; 677.6.845.fastq.gz |
| Bd30-1 | JGI | 1077535 | 2387.4.1909.fastq.gz |
| BdTR1i | JGI | 1079927 | 2152.1.1796.fastq.gz |
| BdTR2b | JGI | 1078982 | 1909.5.1665.fastq.gz; 2041.1.1727.fastq.gz |
| BdTR2g | JGI | 1078866 | 1710.1.1556.fastq.gz |
| BdTR3c | JGI | 1078867 | 1634.4.1486.fastq.gz; 1682.4.1520.fastq.gz; 2358.2.1887.fastq.gz |
| BdTR5i | JGI | 1078863 | 1710.5.1556.fastq.gz |
| BdTR7a | JGI | 1079928 | 2152.5.1797.fastq.gz |
| BdTR9k | JGI | 1078865 | 1710.7.1556.fastq.gz |
| BdTR10c | JGI | 1078868 | 1702.1.1553.fastq.gz |
| BdTR10h | This study | PRJNA377287 | 675_LIB3946_LDI3344_TAGCTT_R1.fastq.gz; |
|  |  |  | 675_LIB3946_LDI3344_TAGCTT_R2.fastq.gz |
| BdTR11a | JGI | 1078983 | 1909.1.1665.fastq.gz |
| BdTR11g | JGI | 1078984 | 1999.3.1704.fastq.gz |
| BdTR11i | JGI | 1078869 | 1999.5.1704.fastq.gz; 1774.3.1601.fastq.gz |
| BdTR13a | JGI | 1079929 | 2152.3.1802.fastq.gz |
| BdTR13c | JGI | 1078861 | 1710.6.1556.fastq.gz |
| BdTR13k | This study | PRJNA377287 | 675_LIB3947_LDI3345_GTGGCC_R1.fastq.gz; |
|  |  |  | 675_LIB3947_LDI3345_GTGGCC_R2.fastq.gz |
| Bis-1 | JGI | 1078977 | 1883.7.1652.fastq.gz; 1957.7.1684.fastq.gz; 2038.2.1726.fastq.gz |
| Foz1 | JGI | 1078874 | 2271.4.1846.fastq.gz |
| Gaz-8 | JGI | 1078864 | 1730.2.1598.fastq.gz |
| Jer1 | JGI | 1078873 | 2388.7.1910.fastq.gz |
| Kah-1 | JGI | 1078980 | 1885.7.1658.fastq.gz; 1957.5.1684.fastq.gz |
| Kah-5 | JGI | 1078981 | 1999.4.1704.fastq.gz |
| Koz-1 | JGI | 1079487 | 2065.7.1739.fastq.gz |
| Koz-3 | JGI | 1077536 | 2385.6.1911.fastq.gz |
| Luc1 | JGI | 1000597 | 6087.6.36752.fastq.gz |
| Mig3 | JGI | 1079485 | 2291.3.1852.fastq.gz |
| Mon3 | JGI | 1079484 | 2263.5.1841.fastq.gz |
| Per1 | JGI | 1000594 | 6087.5.36751.fastq.gz |
| Pob1 | JGI | 1000600 | 6087.7.36753.fastq.gz |
| Sig2 | JGI | 1078876 | 2263.1.1841.fastq.gz |
| Tek-2 | JGI | 1079488 | 2081.3.1743.fastq.gz |
| Tek-4 | JGI | 1078978 | 1883.5.1652.fastq.gz; 1957.6.1684.fastq.gz |
| Uni2 | JGI | 1079486 | 2374.6.1901.fastq.gz |
